# Suppression of HPV-16 late L1 5′-splice site SD3632 by binding of hnRNP D proteins and hnRNP A2/B1 to upstream AUAGUA RNA motifs

**DOI:** 10.1093/nar/gkt803

**Published:** 2013-09-05

**Authors:** Xiaoze Li, Cecilia Johansson, Jacob Glahder, Ann-Kristin Mossberg, Stefan Schwartz

**Affiliations:** Department of Laboratory Medicine, Section of Medical Microbiology, Lund University, 221 84 Lund, Sweden

## Abstract

Human papillomavirus type 16 (HPV-16) 5′-splice site SD3632 is used exclusively to produce late L1 mRNAs. We identified a 34-nt splicing inhibitory element located immediately upstream of HPV-16 late 5′-splice site SD3632. Two AUAGUA motifs located in these 34 nt inhibited SD3632. Two nucleotide substitutions in each of the HPV-16 specific AUAGUA motifs alleviated splicing inhibition and induced late L1 mRNA production from episomal forms of the HPV-16 genome in primary human keratinocytes. The AUAGUA motifs bind specifically not only to the heterogeneous nuclear RNP (hnRNP) D family of RNA-binding proteins including hnRNP D/AUF, hnRNP DL and hnRNP AB but also to hnRNP A2/B1. Knock-down of these proteins induced HPV-16 late L1 mRNA expression, and overexpression of hnRNP A2/B1, hnRNP AB, hnRNP DL and the two hnRNP D isoforms hnRNP D37 and hnRNP D40 further suppressed L1 mRNA expression. This inhibition may allow HPV-16 to hide from the immune system and establish long-term persistent infections with enhanced risk at progressing to cancer. There is an inverse correlation between expression of hnRNP D proteins and hnRNP A2/B1 and HPV-16 L1 production in the cervical epithelium, as well as in cervical cancer, supporting the conclusion that hnRNP D proteins and A2/B1 inhibit HPV-16 L1 mRNA production.

## INTRODUCTION

Human papillomavirus (HPV) is the most common sexually transmitted virus in the human population. Although the vast majority of these HPV infections are cleared by the immune system within a year after infection, HPVs may in rare cases persist and cause cancer (1). HPV is present in 99.7% of all cervical cancers and is tightly associated with several other anogenital cancers and head and neck cancers (2). Nearly half of the human cancers, which are caused by viruses are attributable to HPVs, and cervical cancer is one of the main causes of death in women in the developing world (3). A subset of the sexually transmitted HPV types has been associated with cancer and is termed high-risk HPV types. HPV type 16 is the most common high-risk type in HPV-induced cancers as well as in the human population (4,5).

**Figure 1.**
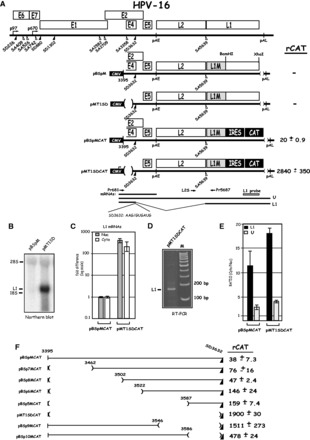
(**A**). Identification of splicing inhibitory sequences immediately upstream of HPV-16 late 5′-splice SD3632. Schematic representation of the HPV-16 genome and the subgenomic HPV-16 expression plasmids. The early and late viral promoters p97 and p670 are indicated. Numbers indicate nucleotide positions of 5′- (filled triangles) and 3′-splice sites (open triangles). The early and late poly (A) sites named pAE and pAL are indicated. L1M represents a previously described mutant HPV-16 L1 sequence in which a number of nucleotide substitutions that inactivate splicing silencers have been inserted downstream of SA5639 (29,32). The sequence of the HPV-16 late 5′-splice site SD3632 is shown. IRES, the poliovirus IRES sequence; CAT, CAT reporter gene; CMV, human cytomegalovirus immediate early promoter; U, unspliced mRNA. Restriction sites BamHI and XhoI used for insertion of IRES and CAT are indicated. mRNAs produced by the plasmids are indicated. The position of the L1 northern blot probe and RT-PCR primers (arrows) are indicated. CAT protein levels produced by each CAT plasmid in the transfected HeLa cells are shown to the right. rCAT was calculated as described in ‘Materials and Methods’ section. Mean values and standard deviations are shown. (**B**) Northern blot on cytoplasmic RNA extracted from HeLa cells transfected with pBSpM or pMT1SD and probed with the L1 probe. (**C**) Real-time PCR of spliced HPV-16 L1 mRNA in nuclear (Nuc) or cytoplasmic (Cyto) fractions of transfected HeLa cells using primers Pr681 and Pr5687 mRNAs as described in ‘Materials and Methods’ section. Graph displays fold difference in L1 mRNA levels between pBSpMCAT and pMT1SDCAT. (**D**) RT-PCR with primers Pr681 and Pr5687 on cDNA of cytoplasmic RNA extracted from HeLa cells transfected with pMT1SDCAT. (**E**) Real-time PCR of HPV-16 spliced L1 mRNAs or unspliced (U) mRNA in nuclear (Nuc) or cytoplasmic (Cyto) fractions of transfected HeLa cells using primers Pr681, L2S and Pr5687 mRNAs as described in ‘Materials and Methods’ section. Graph displays the ratio between cytoplasmic and nuclear L1 or unspliced mRNAs produced from pBSpMCAT and pMT1SDCAT. (**F**) Schematic representations of deletions introduced in HPV-16 subgenomic expression plasmid pBSpMCAT. Plasmid names are shown to the left. Lines represent HPV-16 sequences present in the various plasmids, and numbers indicate ends of deletions. CAT protein levels produced by each plasmid in the transfected cells are shown to the right. rCAT was calculated as described in ‘Materials and Methods’ section. Mean values and standard deviations are shown.

The HPV-16 DNA genome is small, but it contains at least six early genes and two late genes under control of at least two promoters (6). The early promoter p97 is located upstream of the E6 gene, and the late differentiation-dependent promoter named p670 is located upstream of the E1 AUG (7,8). The early region encoding E1, E2, E4, E5, E6 and E7 is followed by the early polyA signal pAE, whereas the late region encodes L1 and L2 and is followed by the late polyA signal pAL (Figure 1A). The HPV-16 life cycle is tightly linked to the differentiation stage of the infected epithelial cell and the late proteins L1 and L2 and viral particles are produced exclusively in terminally differentiated cells, whereas HPV early proteins are produced in the lower and mid layers of the epithelium (9–11).

Alternative splicing is a key mechanism in the control of gene expression of both eukaryotic and viral genes (12–14). A complex pattern of alternatively spliced and polyadenylated mRNAs is observed during the HPV life cycle (15). Both viral early and late mRNAs have multiple introns and exons that contain *cis*-acting RNA elements that interact with cellular splicing factors (16–19). The efficiency by which a splice site is used is determined by *cis*-acting regulatory RNA elements termed splicing enhancers and silencers (20). We have identified a strong splicing enhancer located downstream of HPV-16 3′-splice site SA3358 (19,21,22), the most commonly used splice site on the HPV-16 genome (23–26). Splice site SA3358 is used by both early and late HPV-16 mRNAs and is regulated by ASF/SF2 (SRSF1) (22), SRp30c (SRSF9) (27) and SRp20 (SRSF3) (28). HPV-16 mRNAs that are spliced to SA3358 are either polyadenylated at the early polyA signal pAE or spliced at late 5′-splice site SD3632 to late 3′-splice site SA5639 to produce L1 mRNAs. The latter two splice sites are used exclusively to generate late L1 mRNAs (15). Splice site SA5639 is controlled by multiple splicing silencers located downstream of this site (29,30). These silencers interact with heterogeneous nuclear RNP (hnRNP) A1 (29,31). The inhibitory RNA sequences in the L1 coding region are conserved in many HPV types, including HPV-5, −6b, −16, −18, −31, −45 and −56 (32). These elements strongly suppress L1 expression from any plasmid (33). Indeed, immunization of mice with a plasmid encoding the wild-type L1 gene did not elicit L1 specific antibodies, whereas immunization of mice with an L1 gene in which splicing silencer had been mutationally inactivated was highly immunogenic (34). These results demonstrated that RNA elements efficiently suppress HPV-16 L1 expression. Late 5′-splice site SD3632 is only used for production of spliced L1 mRNAs. It is suppressed by sequences located upstream of SD3632, but these sequences have not been studied in detail (21). Here we report that HPV-16 splice site SD3632 is negatively regulated by two AUAGUA motifs located immediately upstream of SD3632. These sites interacted specifically with members of the hnRNP D family and hnRNP A2/B1. Knock-down of these factors induced HPV-16 late gene expression, whereas overexpression of hnRNP D isoforms hnRNP D37 and hnRNP D40, hnRNP AB, hnRNP DL and hnRNP A2/B1 further suppressed HPV-16 late gene expression. Mutational inactivation of the two AUAGUA motifs induced HPV-16 late gene expression from episomal copies of the HPV-16 genome in primary human keratinocytes. Our results demonstrate that the two AUAGUA motifs regulate HPV-16 late gene expression through interactions with members of the hnRNP D family of proteins and hnRNP A2/B1. We speculate that high levels of hnRNP D proteins observed in the lower layers of cervical epithelium and in cervical cancers, suppress HPV-16 L1 expression in these cells, and that the differentiation-dependent drop in hnRNP D expression seen in cervical epithelium contributes to induction of HPV-16 late gene expression.

## MATERIALS AND METHODS

### Plasmid construction

The following plasmids have been described previously: pBSp (21), pBSpM (35), pMT1SD (35), pOPSDM (21), pTEx4 (22), pTEx4M (22) and pCMVCAT16 (33). We are grateful to Drs Beatrice Orru and Fergus Ryan at Dublin Institute of Technology, Ireland, for generously providing us with plasmids pBELCAT and pBELMCAT (36), to Dr Andras Nagy at University of Toronto, Canada, for providing us with pCAGGS-nlscre (37), to Dr Ethel- Michele de Villiers at Deutsches Krebsforzungszentrum for HPV plasmids, to Dr Oriol Bachs for hnRNP A2/B1 plasmid (38) and to Dr Robert J. Schneider NYU School of Medicine for the hnRNP D plasmids (39). Plasmids encoding the myc-tagged hnRNP AB transcript variant 1 and hnRP DL transcript variant 2 were purchased from OriGene Technologies, Inc., USA.

To generate pBSpCAT and pBSpMCAT, the poliovirus ribosome entry site followed by the chloramphenicol gene (CAT) were inserted between the unique BamHI site in HPV-16 L1 and an XhoI site located immediately after the L1 stop codon in pBSp and pBSpM (35). A fragment containing SD3632 was excised from pMT1SD with HindIII and ApaI and subcloned into pBELMCAT, generating pMT1SDCAT. Previously described plasmids pT5, pT6, pT7, pT8, pT9, pT10 (21) were digested with ApaI and BssHII, and the excised fragments were subcloned into pBSpCAT and pBSpMCAT, generating pBSp5CAT, pBSp6CAT, pBSp7CAT, pBSp8CAT, pBSp9CAT, pBSp10CAT and pBSp5MCAT, pBSp6MCAT, pBSp7MCAT, pBSp8MCAT, pBSp9MCAT, pBSp10MCAT, respectively. pOPSDM was cut with XcmI and HindIII and the fragment encompassing SD3632 was subcloned into pBSpCAT, generating pBSpOPTCAT. An ApaI and BamHI fragment was excised from pBELM (29) and inserted into pBSpOPTCAT to generate pBSpOPTMCAT. To generate pREVSDCAT and pREVSDMCAT, PCR was first carried out on pBELM (29) using oligonucleotides 3455s and 3628as (a list of all oligonucleotides used here is available on request). The amplified fragment was digested with SalI and XbaI and subcloned into pTEx4CAT and pTEx4MCAT, resulting in pREVSDCAT and pREVSDMCAT. QuikchangeII site-directed mutagenesis kit (Agilent technologies) was used to generate pMT4MCAT, pMT5MCAT, pMT6MCAT, pMT7MCAT from pBSp5MCAT with oligonucleotides t5CATM4s and t5CATM4as, t5CATM5s and t5CATM5as, t5CATM6s and t5CATM6as, respectively. Quikchange lightening mutagenesis kit was used to generate pMT1MCAT, pMT2MCAT, pMT3MCAT from pBSp5MCAT with primer t5CATM1S-3, t5CATM2s-4 and t5CATM3s-3 as sense primer, respectively, and M17A as antisense oligonucleotides.

To generate pBSpD1MCAT, pBSpD2MCAT and pBSpD3MCAT, site-directed ligase-independent mutagenesis (SLIM) (40) was performed on pBSp5MCAT by using oligonucleotides BSp5d1MCATFT and BSp5d1MCATFS, BSp5d2MCATFT and BSp5d2MCAT, BSp5d3MCATFT and BSp5d3MCATFS, respectively, and the same BSp5MCATRT, BSp5MCATRS oligonucleotides in all reactions. The same SLIM method was applied to pBSpD1MCAT to generate pMUTD1MCAT, pRMCAT and p880MCAT using oligonucleotides MUTd1MCATFT and MUTd1MCATRT, Md1880MCATFT and Md1880MCATRT, md1RMCATFT and md1RMCATRT respectively, and the same MUTd1MCATFS and MUTd1MCATRS oligonucleotides in all reactions. To generate pM2ATAGTA and pM2ACAC, SLIM mutagenesis was performed on pMUTD1MCAT by using oligonucleotides DOUBLEmut1FT and DOUBLEMUT1RT, DOUBLEMUT2FT and DoubleMUT2RT, respectively, and the same MUTd1MCATFS and MUTd1MCATRS oligonucleotides in both of the reactions. To generate pM1, pM2, pSM2ATAGTA, p4xATAGTA, p4xMUT, SLIM mutagenesis was performed on pBSpD1MCAT by using oligonucleotides mut1D1MCATFT and mut1D1MCATRT, mut2D1MCATFT and mut2D1MCATRT, mut3D1MCATFT and mut3D1MCATRT, mut4D1MCATFT and mut4D1MCATRT, mut9D1MCATFT and mut9D1MCATRT, respectively, in combination with the same MUTd1MCATFS and MUTd1MCATRS oligonucleotides included in all reactions. To generate pME2CAT, pME4CAT, pBSpDCAT, SLIM mutagenesis (40) was performed on pBSpD1MCAT by using oligonuleotides E2MCATFT and E2MCATRT, E4MCATFT and E4MCATRT, BSPDMCATFT and BSPDMCATRT, respectively, in combination with the same MUTd1MCATFS and MUTd1MCATRS oligonucleotides included in all reactions. Plasmids pINWTCAT and pINMUTCAT were generated by PCR amplification of HPV sequences from pBSpMCAT with primers L15SSWT or L15SSMUT and L1BAMAS followed by insertion into pBSpMCAT with SalI and BamHI. To construct pNL13a7HPV16, the HIV-1 sequence between BssHII and HIV-1 5′-splice site SD3 in HIV-1 vpr cDNA pNL13a7 (41) was replaced by the HPV-16 sequence spanning 3546–3628 nt by PCR mutagenesis.

To generate pHPV6MCAT, pHPV18MCAT, pHPVMCAT, pHPV5SMCAT, pHPV41MCAT, SLIM mutagenesis (40) was performed on pBSpD1MCAT by using oligonucleotides HPV6MCATFT and HPV6MCATRT, HPV18MCATFT and HPV18MCATRT, HPVC7910FT and HPVC7910RT, HPV5SFT and HPV5SRT, HPV41FT and HPV41RT, respectively, and the same FS2 and RS2 oligonucleotides in all reactions. Oligonucleotides HPV1FT and HPV1RT, HPV4FT and HPV4RT and HPV5LFT and HPV5LRT, in combination with the same FS3 and RS2 oligonucleotides in all the reactions were used to generate pHVP1MCAT, pHPV4MCAT and pHPV5LMCAT, respectively, using the SLIM method (40) on pBSpD1MCAT. Sense and antisense oligonucleotides 4xWTs and 4xWTas or 4xMUTs and 4xMUTas were annealed and ligated to pUC19T7, generating pUC19T7-4xWT and pUC19T7-4xMUT.

### HPV-16 genomic plasmids

The complete HPV-16 genome was PCR-amplified and inserted into TOPO followed by regeneration of ends upto the unique SphI site. A 34-nt LoxP site was inserted immediately adjacent to the SphI sites flanking the HPV-16 genome. An SphI fragment was deleted from the TOPO vector to generate a plasmid containing only two SphI sites. A sequence encoding the Rous sarcoma virus long terminal repeat followed by the neomycin resistance gene and the simian virus 40 polyA signal was synthesized by Eurofins MWG GmbH. This sequence was inserted downstream of the LoxP site in the genomic HPV-16 plasmid, resulting in pHPV16AN. Introduction of an XhoI site downstream of the L1 stop codon, and insertion of the poliovirus type 2A internal ribosome entry site (IRES) followed by the gene encoding the secreted luciferase (sLuc) (42) between the unique BamHI- and XhoI-sites, resulted in the pHPV16ANsL plasmid. pHPV16MANsL was generated by insertion of a ApaI-BamHI fragment from pBELM (29) into pHPV16ANsL. To generate mutants in the silencer at SD3632, mutagenesis was performed on a SexA1-ApaI fragment of the HPV-16 genome using the primers described earlier in the text. Mutagenesis was performed with the SLIM method, (40) and all mutants were sequenced before and after insertion into pHPV16ANsL or pHPV16MANsL, resulting in p2xmut, and pME4 and pM2mut, respectively.

### Transfection of HeLa and C33A2 cells

The C33A2 cell line is a C33A derived cell line that contains the pBELsluc reporter plasmid stably integrated into the genome, as described previously for pBELCAT in HeLa cells (36). The pBELsluc produces primarily spliced E4 mRNAs, and late gene expression is undetectable due to suppression of late splice sites SD3632 and SA5639. Late gene expression can be induced, which results in production of sLuc in the cell culture medium. The C33A2 cell line will be described in detail elsewhere. HeLa and C33A2 cells were cultured in Dulbecco’s modified Eagle medium with 10% heat-inactivated fetal bovine calf serum and penicillin-streptomycin. Transfections were carried out using Turbofect according to the manufacturer’s instructions (Fermentas). Briefly, the mixture of 2 µl of Turbofect and 100 µl of Dulbecco’s Modified Eagle medium without serum was added to 1µg of plasmid DNA and incubated at room temperature for 15 min before dropwise addition to 60-mm plates with subconfluent HeLa cells. Cells were harvested at 24 h posttransfection. Each plasmid was transfected in triplicate, in a minimum of two independent experiments.

### Propagation and transfection of human primary keratinocytes

Neonatal human epidermal keratinocytes were purchased from Gibco Invitrogen Cell Culture and were propagated in EpiLife Medium supplemented with Human keratinocyte Growth Supplement (Gibco Invitrogen Cell Culture) as recommended by the manufacturer. The primary keratinocytes are grown in monolayer cultures and expanded under growth conditions that maintain poorly differentiated cells with properties similar but not identical to the cells in the basal layers of the epithelium. Cells passaged less than three times were used. In all, 150 000 cells were seeded per 60-mm plate for each transfection. Co-transfections of genomic HPV-16 plasmids and plasmid pCAGGS-nlscre (37) (generously provided by Dr Andras Nagy at University of Toronto), which expresses the cre recombinase, were carried out using Fugene 6 (Roche), as previously described for HPV-18 (43,44). Briefly, the mixture of 9 µl of Fugene 6 and 200 µl of EpiLife Medium with supplements was added to 3 µg of plasmid DNA and incubated at room temperature for 15 min before dropwise addition to 60-mm plates with subconfluent neonatal human epidermal keratinocytes cells. At 24 h after transfection, G418 was added to the cells at a concentration of 100 µg/ml. Medium was harvested for analysis of sLuc at different time points after transfection. Total RNA or DNA analysis was performed at day 6 posttransfection. Each plasmid was transfected in triplicates, in a minimum of two independent experiments.

### CAT enzyme-linked immunosorbent assay

Cell extracts were prepared from transfected HeLa cells at 24 h posttransfection according to the protocol from a CAT enzyme-linked immunosorbent assay (ELISA) kit (Roche). All cell extracts were appropriately diluted to obtain an absorbance reading in the linear range of the CAT ELISA assay. To be able to compare CAT results between experiments, pCMVCAT16 was transfected in triplicate in each transfection experiment. Arbitrary CAT units were calculated by multiplying the absorbance 405 readings with the dilution factor for each sample. CAT units obtained for each sample were divided by CAT units obtained in pCMVCAT16 transfections and thereafter multiplied by a factor of 10^4^ to obtain the rCAT units displayed in the manuscript figures. Mean values and standard deviations were calculated for all samples.

### sLuc assay

The *Metridia longa* sLuc (42) activity in the medium of the transfected cells was monitored with the help of the Ready To Glow Secreted Luciferase Reporter assay according the instructions of the manufacturer (Clontech). Briefly, 50 µl of cell culture medium was added to 5 µl of 0.5X Secreted Luciferase substrate/Reaction buffer in a 96-well plate and luminiscence was determined in a Tristar LB941 Luminometer.

### DNA extraction

Cellular DNA was extracted after lysis of cells in lysis buffer [10 mM Tris–HCl (pH 8.0)-buffer supplemented with 100 mM NaCl, 25 mM EDTA, 0.5% SDS and 250 µg proteinase K]. The cell extracts were incubated at 50°C over night and an equal volume of phenol/chloroform/isoamyl alchohol (chisam) was added. The aqueous phase was collected after centrifugation of the samples and subjected to ethanol precipitation. The samples were treated with RNaseA and stored at −20°C until use. To monitor recombination at the loxP sites in pHPV16AN and pHPV16ANsL, PCR was performed with primers 16S (5′-TATGTATGGTATAATAAACACGTGTGTATGTG-3′) and 16A (5′-GCAGTGCAGGTCAGGAAAACAGGGATTTGGC-3′) (see Figure 4A). This PCR reaction yields a 366-nt PCR fragment that is diagnostic for recombination at the LoxP sites.

### RNA extraction, reverse transcription-PCR and northern blotting

Cytoplasmic RNA was extracted 24 h posttransfection using NP-40 buffer as described previously (35). Total RNA was extracted by Tri reagent (Sigma) according to manufacturer’s protocol. Two hundred nanograms of cytoplasmic RNA were reverse transcribed in 25 ul reaction at 42°C by using Superscript II and random hexamers according to the protocol of the manufacturer (Invitrogen). Two microliter of cDNA was subjected to PCR amplification or real-time PCR. Real-time PCR was performed in a MiniOpticon (BioRad) using the Sso Advanced SYBR Green Supermix (BioRad) according the manufacturers’ instructions. PCR-primer sequences are available on request. The northern blot analysis was carried out by size separation of 5 µg of cytoplasmic RNA on a 1.2% agarose gels containing 2.2 M formaldehyde. The RNA was transferred overnight onto nitrocellulose filters and hybridized to L1 probe. The L1 probe was excised from pBEL with BamHI and XhoI and radiolabeled with ^32^P-CTP, as described previously (35).

### *In vitro* transcription and ultraviolet cross-linking

PCR fragments amplified from plasmids pUC19T7-4xWT and pUC19T7-4xMUT with oligonucleotides T7s and 4xWTas or 4xMUTas, respectively, were transcribed *in vitro* with T7-RNA polymerase (Ambion) according to manufacturer’s instructions. Briefly, 1 µg of DNA and T7-RNA polymerase (50 U) were incubated at 37°C for 4 h with 50 U Riboblock RNase inhibitor (Fermentas), 10 µl of transcription buffer (Ambion), 40 uCi of ^32^P-UTP (3000 Ci/mmol) (Perkin Elmer) in a total volume of (100 µl) in the presence of 2 mM each of rATP, rCTP and rGTP. The samples were treated with RNase-free DNase (Fermentas) at 37°C for 30 min, extracted with phenol-chloroform, and ethanol precipitated. The radiolabeled RNAs were pelleted and dissolved in 50 µl of RNase-free distilled water. The unlabeled RNA competitors were transcribed under similar conditions, except that the radiolabeled nucleotide was substituted with 1 mM UTP. The unlabeled transcripts were dissolved in RNase-free distilled water to a concentration of ∼2 µg/µl. The quality of the probes or RNA competitors was examined by electrophoresis on 8% urea polyacrylamide gels. RNAs were visualized by Gel-red staining of gels or by autoradiography.

Ultraviolet (UV) cross-linking was carried out as described previously (29). Briefly, cell extracts were incubated with radiolabeled RNA in binding buffer [10 mM HEPES (pH 7.6), 60 mM KCl, 3 mM MgCl_2_, 1 mM DTT, 5% glycerol and 5 µg/µl heparin] for 15 min at room temperature followed by UV irradiation in a Bio-link cross-linker (Biometra) for 15 min. For competition experiments, cell extract was pre-incubated with the RNA competitors in the binding buffer for 10 min before addition of the radiolabeled RNA probe. After UV irradiation, RNaseA (20 µg) and RNaseT1 (1000U) were added to the samples that were incubated for 30 min at 37°C. The UV cross-linked RNA-proteins were resolved on 12% SDS–PAGE gels, and the bands were visualized by autoradiography.

### RNA-protein pull-downs followed by western blotting or mass spectrometry

Sixty micrograms of HeLa nuclear extract was mixed with streptavidin-coated magnetic beads bound to the various biotin-labeled RNA oligonucleotides indicated in the figures in 200 µl of binding buffer [10 mM Tris (pH 7.4), 50 mM NaCl, 2.5 mM MgCl_2_, 0.5% Triton X-100]. After incubation with rotation for 20 min at room temperature, beads were washed 10 times in binding buffer with 200 mM NaCl. Proteins were eluted by boiling beads in SDS–PAGE loading buffer. After electrophoresis, the gels were either subjected to western blotting as described previously (45) using antibodies indicated in the figures (anti-hnRNP DL antibody (Abcam83215), anti-hnRNP D antibody (Abcam61193), anti-hnRNP A2/B1 antibody (Abcam31645), anti-KHSRP antibody (Abcam150393) or anti-hnRNP AB antibody (Sigma, SAB2701788), or stained by Silver statin (SilverQuest™ staining Kit, Invitrogen), followed by excision of bands and liquid chromatography-mass spectrometry (LC-MS) analysis at the SCIBLU Proteomics Resource Centre at Lund university.

### Small interfering RNA knock downs

All small interfering RNA (siRNA) was purchased from Qiagen, and all siRNAs were transfected into C33A2 cells using RNAiFect™ transfection reagent (Qiagen) according to the manufacture’s protocol. Medium was replaced at 24 h posttransfection, and sLuc activity was monitored at 96 h posttransfection. Cells were either lysed in RIPA buffer [50 mM Tris (pH 7.6), 150 mM NaCl, 0.1% SDS, 0.5% Na-deoxycholate, 1% NP40] followed by western blotting as previously described (45) with antibodies indicated in the figures or were used for preparation of RNA as described earlier in the text that was used for real-time PCR with primers 757s and L1A.

## RESULTS

### A 232-nt deletion upstream of HPV-16 late 5′-splice site SD3632 induced L1 mRNA expression

HPV-16 splice sites SD3632 and SA5639 are used exclusively to produce the late L1 mRNAs (Figure 1A) (15). To investigate how SD3632 is regulated, we constructed subgenomic HPV-16 plasmid pBSpM that contains both splice sites, but lacks early splice sites. Although splicing silencers at late 3′-splice site SA5639 are mutationally inactivated (L1M, Figure 1A) (29), this plasmid fails to produce detectable levels of spliced L1 mRNA on transfection into HeLa cells, as SD3632 is suppressed (Figure 1B). However, deletion of a 232-nt sequence upstream of SD3632 as in pMT1SD (Figure 1A) resulted in a high expression of spliced L1 mRNAs (Figure 1B). We concluded that sequences immediately upstream of HPV-16 late 5′-splice site SD3632 inhibit production of spliced L1 mRNA.

To facilitate further studies of the splicing regulatory RNA sequences upstream of SD3632, we inserted the CAT reporter gene in the L1 region to measure CAT as a quantitative marker for L1 mRNA production. Poliovirus IRES and CAT were inserted between the BamHI site, located 518 nt downstream of the L1 AUG, and an XhoI site at the L1 stop codon, in the two plasmids pBSpM and pMT1SD described earlier in the text (Figure 1A), to generate plasmids pBSpMCAT and pMT1SDCAT (Figure 1A). We transfected pBSpMCAT and pMT1SDCAT in triplicates into HeLa cells and measured CAT levels by CAT ELISA. In all transfection experiments, the positive control plasmid pCMVCAT16 was also transfected in triplicates. CAT units obtained in transfections with various HPV-16-CAT plasmids were divided by the CAT units produced by pCMVCAT16 and thereafter multiplied by 10^4^ to generate the rCAT values displayed in each figure, as described in ‘Materials and Methods’ section. A comparison between pBSpMCAT and pMT1SDCAT revealed that deletion of 232 nt immediately upstream of SD3632 in pBSpMCAT, as in pMT1SDCAT (Figure 1A), resulted in a >100-fold increase in CAT production (Figure 1A), supporting the idea that inhibitory RNA sequences are present in the 232 nt located immediately upstream of SD3632. Analysis of spliced L1 mRNA levels in the nucleus as well as in the cytoplasm of HeLa cells transfected with pBSpMCAT or pMT1SDCAT revealed that pMT1SDCAT produced >100-fold more spliced L1 mRNA than pBSpMCAT (Figure 1C). As this difference was observed also in the nucleus, the results suggested that the sequence upstream of SD3632 inhibited mRNA splicing. Cloning and sequencing of reverse transcription (RT)-PCR products from pMT1SDCAT-transfected cells (Figure 1D) confirmed that the mRNAs produced from pMT1SDCAT used HPV-16 late splice sites SD3632 and SA5639. Furthermore, analysis of the ratios of cytoplasmic versus nuclear L1 mRNAs, as well as the unspliced (U) mRNAs produced by pBSpMCAT or pMT1SDCAT demonstrated that mRNAs containing the exonic splicing inhibitor element (mRNAs produced from pBSpMCAT) were exported from the nucleus to the cytoplasm with similar efficiency as those lacking the inhibitory element (mRNAs produced from pMT1SDCAT) (Figure 1E). Although the cytoplasmic versus nuclear ratio was higher for pMT1SDCAT-derived mRNAs compared with pBSpMCAT-derived mRNAs (Figure 1E), it was relatively minor compared with the effect on splicing. Taken together, these results established that splicing inhibitory sequences are located upstream of SD3632 and validated the use of the CAT reporter plasmids as a tool to investigate HPV-16 L1 mRNA splicing.

### HPV-16 splice site SD3632 is suboptimal

We optimized HPV-16 SD3632 by changing this 5′ splice site to a sequence with perfect complementary to U1 snRNA (21) in plasmids pBSpCAT and pBSpMCAT, generating pBSpOPTCAT and pBSpOPTMCAT, respectively (Supplementary Figure S1A). Plasmid pBSpOPTCAT produced 21 times more CAT than pBSpCAT (Supplementary Figure S1B). pBSpMCAT, in which also splicing silencers at SA5639 had been inactivated, produced 30-fold more CAT than pBSpCAT (Supplementary Figure S1B). These results confirmed that both SD3632 and SA5639 were subject to negative regulation, and established that SD3632 is suboptimal. Plasmid pBSpOPTMCAT, in which SD3632 had been optimized and splicing silencers at SA5639 had been destroyed, produced 499-fold higher CAT levels than pBSpCAT (Supplementary Figure S1B). However, these CAT levels were >6-fold lower than those produced by pMT1SDCAT (Supplementary Figure S1B), indicating that the splicing inhibitory sequence upstream of SD3632 also inhibited the optimized SD3632, but to a lower extent than wild-type SD3632. The combined effect of optimization of 5′-splice site SD3632 and mutational inactivation of splicing silencers at SA5639 was several fold higher than optimization of each splice site alone, which suggested that splicing silencers at SD3632 or SA5639 acted independently of each other.

### Splicing inhibitory sequences are located in the 34 nt immediately upstream of HPV-16 late 5′-splice site SD3632

To map the location of the inhibitory RNA elements upstream of HPV-16 SD3632, we generated serial deletions in this region in pBSpMCAT, resulting in plasmids pBSp5MCAT, pBSp6MCAT, pBSp7MCAT and pBSp8MCAT (Figure 1F). All plasmids expressed substantially lower CAT levels than pMT1SDCAT (Figure 1F). We concluded that pBSp5MCAT contained the smallest inhibitory element, and that the splicing inhibitory element is located in the 42 nt between HPV-16 genomic position 3587 and the late 5′-splice site SD3632 (Figure 1F). To confirm these results, we also introduced internal deletions in this region, generating plasmids pBSp9MCAT and pBSp10MCAT, in which sequences containing the inhibitory RNA elements between position 3547 and 3628 had been deleted (Figure 1F). Both plasmids produced high levels of CAT (Figure 1F), which confirmed that the 42 nt upstream of SD3632 contained a major splicing inhibitory element. The ∼3-fold lower CAT production from pBSp10MCAT compared with pBSp9MCAT, indicated that additional splicing regulatory elements were located upstream of nucleotide position 3587, but they inhibited splicing to a lower extent.

We also inserted the same deletions in pBSpCAT, in which splicing silencers at SA5639 were intact, generating pBSp5CAT, pBSp6CAT, pBSp7CAT, pBSp8CAT pBSp9CAT and pBSp10CAT (Supplementary Figure S1C). CAT production from pBSpCAT-derived plasmids was overall lower than from pBSpMCAT-derived plasmids (Supplementary Figure S1D). The results confirmed that splicing inhibitory RNA elements at SD3632 are located within the 42 nt immediately upstream of HPV-16 SD3632 (Supplementary Figure S1D), and that additional but weaker splicing regulatory elements are present upstream of nucleotide position 3587. We concluded that the major splicing inhibitory element was located between nucleotide position 3587 and SD3632. These sequences were studied further.

Next we investigated whether the splicing of HPV-16 SD3632 could function in a heterologous context. The sequence immediately upstream of HPV-16 SD3632 (3546–3628 nt) was therefore inserted immediately upstream of HIV-1 5′-splice site SD3 in the HIV-1 vpr cDNA expression plasmid pNL13a7 (Supplementary Figure S2A) (41). The effect of the HPV-16 splicing silencer on the splicing from HIV-1 SD3 to SA4 was monitored by RT-PCR, and the results revealed that the sequence immediately upstream of HPV-16 late 5′-splice site SD3632 could inhibit HIV-1 5′-splice site SD3 (Supplementary Figure S2B). Overexpression of the splicing inhibitory factor PTB was performed to show production of the unspliced 13a7 vpr mRNA from pNL13a7.

**Figure 2.**
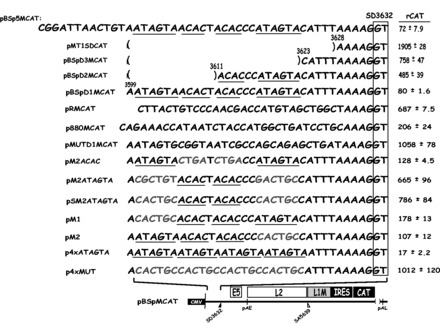
Two nucleotide substitutions in each AUAGUA motif inactivate the splicing suppressors at HPV-16 late 5′-splice site SD3632. Schematic representations of subgenomic HPV-16 expression plasmids. Plasmid names are shown to the left. HPV-16 sequences present in the various plasmids are shown. Nucleotides in red indicate mutated positions. The AUAGUA and ACAC motifs are underlined. Filled and empty triangles represent late 5′-splice site SD3632 and late 3′-splice site SA5639. Early and late poly (A) signals pAE and pAL are indicated. L1M, a mutant L1 sequence in which splicing silencers downstream of SA5639 had been inactivated (29,32); CMV, human cytomegalovirus immediate early promoter; IRES, the poliovirus IRES sequence; CAT, CAT reporter gene. CAT protein levels produced by each plasmid in the transfected cells are shown to the right. rCAT was calculated as described in ‘Materials and Methods’ section. Mean values and standard deviations are shown.

To map the inhibitory RNA elements further, three additional deletions were inserted into pBSp5MCAT (Figure 2). These deletion mutants were termed pBSpD1MCAT, pBSpD2MCAT and pBSpD3MCAT (Figure 2). They contain 12 nt consecutive deletions from the 5′-end in pBSp5MCAT (Figure 2). Plasmid pBSpD1MCAT, which contained 34 nt upstream of SD3632, expressed similar levels of CAT as pBSp5MCAT, whereas plasmids pBSpD2MCAT and pBSpD3MCAT produced elevated levels of CAT (Figure 2). We concluded that pBSpD1MCAT contained the smallest splicing inhibitory sequence and that this sequence consisted of 34 nt immediately upstream of SD3632, between HPV-16 genomic positions 3599 and 3632 (Figure 2).

### Two AUAGUA sequences inhibit HPV-16 late 5′-splice site SD3632

To investigate the sequence specificity of the splicing inhibitory sequence, we replaced this 34-nt sequence with a computer-randomized 34-nt sequence, resulting in pRMCAT (Figure 2). In addition, we replaced the 34-nt upstream of SD3632 with the corresponding 34 nt located upstream of the HPV-16 early splice site SD880, resulting in p880MCAT (Figure 2). HPV-16 SD880 is fully active in cervical cancer cells and is therefore not under negative regulation in HeLa cells. High levels of CAT were expressed from both p880MCAT and pRMCAT compared with pBSpMCAT and pBSpD1MCAT (Figure 2). We concluded that the 34 nt located between 3599 and 3632 immediately upstream of HPV-16 late splice site SD3632 contained splicing inhibitory elements that acted in a sequence specific manner to inhibit SD3632.

Two sequence motifs are repeated twice within the 34 nt upstream of SD3632: ACAC and AUAGUA (underlined in pBSpD1MCAT in Figure 2). To investigate whether these RNA motifs contributed to inhibition of SD3632, we introduced nucleotide substitutions in all ACAC and AUAGUA motifs resulting in pMUTD1MCAT (Figure 2). This plasmid produced high levels of CAT (Figure 2), demonstrating the inhibitory element had been inactivated. Plasmid pM2ACAC (Figure 2), in which both ACAC motifs had been replaced by CUGA, produced CAT levels similar to those produced by pBSpD1MCAT (Figure 2), demonstrating that splicing silencers were largely unaffected by these mutations. In contrast, plasmid pM2ATAGTA (Figure 2), in which the two AUAGUAs had been replaced by CGCUGU or ACUGCC, or plasmid pSM2ATAGTA, in which the two AUAGUAs had been replaced by the same CACTGC sequence, produced high levels of CAT (Figure 2). We concluded that one or both AUAGUA motifs inhibited splicing of HPV-16 late 5′-splice site SD3632.

To determine whether both AUAGUA motifs contributed to inhibition of SD3632, they were individually mutated by replacing each AUAGUA motif with CACUGC, resulting in pM1 and pM2 (Figure 2). Both plasmids produced low levels of CAT, indicating that both AUAGUA motifs inhibited SD3632, and that they acted independently of each other (Figure 2). To provide further evidence for the importance of the AUAGUA motifs, we inserted four copies of the wild-type AUAGUA sequence, or of the mutant CACUGC sequence, upstream of SD3632, resulting in plasmids p4xATAGTA and p4xMUT, respectively (Figure 2). Plasmid p4xATAGTA produced low levels of CAT (Figure 2), whereas p4xMUT produced high levels of CAT (Figure 2). Thus, four AUAGUA motifs efficiently inhibited splicing from SD3632, whereas four copies of the mutant sequence did not. These results established that AUAGUA motifs can inhibit HPV-16 late 5′-splice site SD3632.

**Figure 3.**
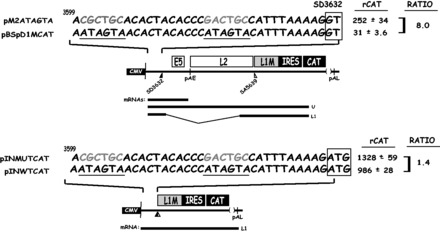
The splicing silencer at HPV-16 5′-splice site SD3632 primarily inhibits production of spliced HPV-16 L1 mRNAs. The mutant or wild-type splicing silencer was inserted in a ‘pre-spliced’ L1 mRNA made from pBSpMCAT generating plasmids pINMUTCAT and pINWTCAT to monitor the effect of the silencer on an L1 mRNA that cannot be spliced. The effect was compared with the CAT protein levels produced from plasmids pM2ATAGTA and pBSpD1MCAT that contain the same wild-type and mutant splicing silencer sequences as the ‘pre-spliced’ mRNAs produced from produced pINMUTCAT and pINWTCAT. CAT levels produced by each plasmid in the transfected cells are shown to the right. rCAT was calculated as described in ‘Materials and Methods’ section. Mean values and standard deviations are shown. The ratio between the CAT levels produced from mutant and wild-type plasmids are shown to the right. CMV, human cytomegalovirus immediate early promoter; IRES, the poliovirus IRES sequence; CAT, CAT reporter gene. mRNAs produced by the HPV plasmids are indicated.

To provide further evidence for the AUAGUA motifs acting at the level of splicing rather than stability or mRNA transport, we constructed a pair of plasmids that monitored the effect of the splicing silencer element on ‘pre-spliced’ mRNAs. The mutant or wild-type splicing silencer sequence in plasmids pM2ATAGTA and pBSpD1MCAT were inserted into a plasmid that produces a ‘pre-spliced’ L1 mRNA that cannot be spliced further (Figure 3). This resulted in plasmids pINMUTCAT and pINWTCAT (Figure 3). The effect of the mutant and wild-type sequences on CAT production was determined and compared with the effect on CAT production of the same mutant and wild-types sequences from pM2ATAGTA and pBSpD1MCAT that produce mRNAs that can splice. The results revealed an 8-fold difference in CAT production between pM2ATAGTA and pBSpD1MCAT, whereas there was only a 1.4-fold difference between pINMUTCAT and pINWTCAT (Figure 3). Although the presence of the splicing silencer motifs on mRNAs that lack the capacity to splice may hinder efficient nuclear export or reduce mRNA stability to some extent, we concluded that the major effect of the AUAGUA motifs was on the mRNA splicing process.

### Regulation of late 5′-splice site SD3632 by AUAGUA motifs is not conserved in the papillomavirus family

We compared the 34-nt sequence upstream of HPV-16 late 5′-splice site SD3632 with the corresponding sequence within different HPV genera, including: HPV-6, HPV-18 (Alpha genus), HPV-5 (Beta genus), HPV-4 (Gamma genus), HPV-1 (Mu genus) and HPV-41 (Nu genus) (Supplementary Figure S3) (46). Analysis of the various sequences revealed that the two AUAGUA motifs were poorly conserved, if at all (Supplementary Figure S3). Only HPV-16 contained two identical copies of AUAGUA, while other members of species nine alpha viruses contained different, but closely related sequence motifs, i.e. AUAGUG or AUAAUA instead of AUAGUA (Supplementary Figure S3). A consensus of all species 9 HPVs of the alpha genus revealed that the consensus of the proximal AUAGUA motif was identical to the HPV-16 sequence, whereas the distal motif was not (data not shown). The consensus of the proximal AUAGUA motif in species 7, 9 and 10 of the alpha genus was also AUAGUA, as seen in pHPVMCAT (Supplementary Figure S3). In contrast, the distal HPV-16 motif was not conserved (Supplementary Figure S3). We concluded that the presence of two AUAGUA motifs upstream of late 5′-splice site SD3632 appears to be unique for HPV-16. This is of interest in the light of the fact that HPV-16 sticks out as the most common HPV type in the human population and as such must be endowed with unique properties.

We replaced the 34 nt upstream of HPV-16 SD3632 in pBSpD1MCAT with sequences of the corresponding region in HPV-6, HPV-18, HPV-41, HPV-1, HPV-4 and HPV-5, generating pHPV6MCAT, pHPV18MCAT, pHPV41MCAT, pHPV1MCAT pHPV4MCAT, pHPV5SMCAT and pHPV5LMCAT (Supplementary Figure S3). The 34-nt sequences of pHPV5SMCAT and pHPV5LMCAT were obtained from two differentially spliced cDNAs (15). We also inserted a 34-nt sequence representing the consensus sequence of species 7, 9 and 10 (alpha genus), generating pHPVMCAT (Supplementary Figure S3). High levels of CAT were produced from all plasmids except pHPV5LMCAT, pHPV1MCAT, pHPVMCAT and HPV-16 plasmid pBSpD1MCAT (Supplementary Figure S3). Plasmids pHPV6MCAT and pHPV18MCAT encode sequences that are related to the AUAGUA motifs: AUAGUG motif in HPV6 and GUGGUA and AUAAUA motifs in HPV18, but these motifs did not inhibit splice site SD3632 to the same extent as the two AUAGUA motifs in HPV-16 (Supplementary Figure S3). The pHPVMCAT plasmid that contained an AUAGUA motif at the proximal position and a closely related GUUGUA motif at the distal position produced low levels of CAT (Supplementary Figure S3), supporting the importance of the AUAGUA sequence. Sequences of the cutaneous types HPV-1 and HPV-5L also inhibited HPV-16 SD3632, but these viral sequences did not encode AUAGUA motif, indicating that other sequences may inhibit splicing. Both HPV-1 and HPV-5L sequences are G-rich, a property of previously described splicing silencers that interact with hnRNP H (47). We concluded that AUAGUA motifs have an inhibitory effect on HPV-16 SD3632 and that the presence of two copies of this sequence upstream of the late 5′-splice site appears to be unique to HPV-16.

### Two nucleotide substitutions in each AUAGUA motif inactivate the splicing suppressors at HPV-16 late 5′-splice site SD3632

**Figure 4.**
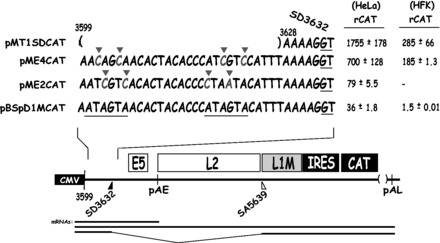
Two nucleotide substitutions in each AUAGUA motif inactivate the splicing suppressors at HPV-16 late 5′-splice site SD3632. Schematic representations of subgenomic HPV-16 expression plasmids. Plasmid names are shown to the left. HPV-16 sequences present in the various plasmids are shown. Nucleotides in red marked with red triangles indicate mutated positions. The AUAGUA motifs are underlined. Filled and empty triangles represent late 5′-splice site SD3632 and late 3′-splice site SA5639. Early and late poly (A) signals pAE and pAL are indicated. L1M, a mutant L1 sequence in which splicing silencers downstream of SA5639 had been inactivated (29,32); CMV, human cytomegalovirus immediate early promoter; IRES, the poliovirus IRES sequence; CAT, CAT reporter gene. mRNAs produced by the HPV-16 plasmids are indicated. CAT protein levels produced by each plasmid in transfected HeLa cells or HFKs cells are shown to the right. rCAT was calculated as described in ‘Materials and Methods’ section. Mean values and standard deviations are shown.

The mutations in the HPV-16 AUAGUA motifs described earlier in the text also affected the overlapping E2 and E4 genes. As both E2 and E4 open reading frames (ORFs) overlap the AUAGUA motifs, it is impossible to mutate the AUAGUA motifs without also changing either the E2 or the E4 coding sequence. We therefore constructed two more mutants of the AUAGUA motifs that either affected the E2 ORF or the E4 ORF, resulting in plasmids pME2CAT and pME4CAT, respectively (Figure 4). In pME4CAT, the two T to C substitutions in the distal AUAGUA motif and the two A to C substitutions in the proximal AUAGUA motif, resulted in a I to T and a V to A amino acid substitution in the distal motif, and inactivation of the E4 stop codon in the proximal motif, which prolonged the E4 ORF with three amino acids. None of these mutations affected the E2 protein sequence. In plasmid pME2, two A to C substitutions were introduced in the distal AUAGUA motif, and an A to C and a G to A substitution were introduced in the proximal AUAGUA motif. This resulted in four amino acid substitutions in the E2 ORF (S to R, N to H, I to L and V to I), whereas the E4 protein sequence was intact. Neither E2 nor E4 can be expressed from these plasmids. High levels of CAT were produced from pME4CAT, whereas pME2CAT produced CAT levels similar to those produced by the wild-type pBSpD1MCAT plasmid (Figure 4). We concluded that the 2-nt substitution in each AUAGUA motif in pME4 inactivated the inhibitory element upstream of HPV-16 SD3632, whereas the nucleotides substitutions in pME2 did not. Deletions of the entire region as in pMT1SDCAT (Figure 4), also resulted in high CAT expression, as expected (Figure 4). Similar results were obtained by transfection of primary human keratinocytes, albeit with overall lower levels of CAT owing to lower transfection efficiency of these cells compared with HeLa cells (Figure 4). The primary human foreskin keratinocytes (HFK) were grown in monolayer cultures and expanded under growth conditions that maintain poorly differentiated cells with properties similar but not identical to the cells in the basal layers of the epithelium, cells in which HPV-16 late gene expression is normally suppressed. In conclusion, the splicing silencer at HPV-16 late 5′-splice site SD3632 is active in primary human keratinocytes, and the 4-nt substitutions in pME4 were sufficient to inactivate this splicing silencer.

### The two AUAGUA motifs inhibit late L1 mRNA splicing in the full-length episomal HPV-16 genome expressed in human primary keratinocytes

**Figure 5.**
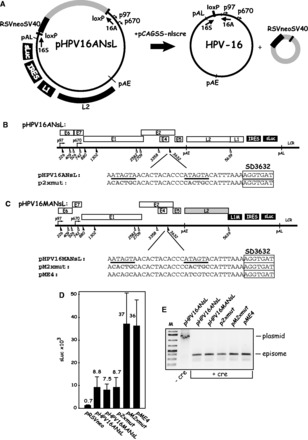
(**A**). The two AUAGUA motifs inhibit late L1 mRNA splicing in the full-length, episomal HPV-16 genome expressed in human primary keratinocytes. Structure of the HPV-16 genomic plasmids pHPV16ANsL. LoxP sites and HPV-16 early (p97) and late (p670) promoters are indicated. The cassette encoding the Rous sarcoma virus long terminal repeat promoter driving the neomycin resistance gene, followed by the simian virus 40 polyA signal is indicated (RSVneo). Arrows indicate positions of PCR primers 16S and 16A. The effect of the cre recombinase produced by the cotransfected plasmid pCAGSS-nlscre on these plasmids is illustrated. pAE and pAL, HPV-16 early and late polyA signals, respectively; L1 and L2, late HPV-16 genes L1 and L2; sLuc, sLuc (42); IRES, poliovirus 2A IRES. (**B, C**) Schematic representation of the HPV-16 genomic plasmids pHPV16ANsL and pHPV16MANsL. The early and late viral promoters p97 and p670 are indicated. Numbers indicate nucleotide positions of 5′- (filled triangles) and 3′-splice sites (open triangles). The early and late poly (A) sites pAE and pAL are indicated. The mutations introduced in the splicing silencer at late 5′-splice site SD3632 are indicated in red and the AUAGUA motifs are underlined. The names of the mutant plasmids are shown to the left. L1M represents a previously described mutant HPV-16 L1 sequence in which a number of nucleotide substitutions that inactivate splicing silencers have been inserted downstream of SA5639 (29,32). IRES, the poliovirus IRES sequence; sLuc, secreted luciferase gene (42); LCR, long control region. (**D**) sLuc activity in cell culture medium collected at day 5 posttransfection of human primary keratinocytes transfected with the indicated HPV-16 plasmids. Plasmids were cotransfected with pCAGGS-nlscre (37) to produce the episomal form of the HPV-16 genomes. (**E**) PCR with primers 16S and 16A on Hirt DNA extracted from human primary keratinocytes transfected with the indicated HPV-16 plasmids in the presence of the cre-expressing plasmid pCAGGS-nlscre (37). Primers 16S and 16A are located on each side of the LoxP sites in the HPV-16 plasmids and the PCR-reaction yields a 366-nt PCR fragment that is diagnostic for recombination at the LoxP sites. A larger band is amplified from plasmid DNA that has not recombined (−cre). M, molecular size marker.

We wished to determine whether the two AUAGUA motifs were active in the context of the HPV-16 genome and in human primary keratinocytes. We therefore generated genomic plasmid pHPV16ANsL, in which a complete HPV-16 genome is flanked by loxP-sites and is released to its episomal form on transfection of pHPV16ANsL with plasmid pCAGGS-nlscre (37), as previously described for HPV-18 (44). To easily monitor and quantitate differences in HPV-16 late gene expression, L1 sequences from the BamHI site located 518 nt downstream of the L1 ATG to the L1 stop codon were replaced by the poliovirus IRES followed by the sLuc gene (42) (Figure 5A). Effects of various mutations on HPV-16 late gene expression could then be determined by the quantitation of sLuc in the medium of the transfected cells. To investigate whether the two AUAGUA motifs inhibited HPV-16 late gene expression also in the context of the viral genome, we mutated splicing silencers at either SD3632, SA5639 or at both splice sites in pHPV16ANsL (Figure 5B). If the splicing silencers at these splice sites play a role in HPV-16 gene expression in keratinocytes, and in the context of the viral genome, the mutants should produce elevated levels of sLuc, as a result of enhanced late gene expression. We replaced the two AUAGUA motifs at SD3632 with the CACUGC sequence in pHPV16ANsL, resulting in p2xmut (Figure 5B). Although the introduced mutations targeted silencers at SD3632, splicing from SD3632 to SA5639 would not occur efficiently, as splice acceptor SA5639 is suppressed by multiple splicing silencers in the L1 coding region (29,30). We therefore also introduced the same mutations in pHPV16MANsL, in which also the splicing silencers at SA5639 had been inactivated, resulting in pM2xmut (Figure 5C). The mutations in L1 affected the L1 DNA and RNA sequences, but not the L1 protein sequence (32). The wild-type and mutant HPV16 genomes were transfected into primary keratinocytes in the presence of pCAGGS-nlscre (37), and the levels of s-Luciferase were monitored at day 5 posttransfection (Figure 5D). HPV-16 genomes with mutations in either the silencers at SD3632 (p2xmut) or the silencers at SA5639 (pHPV16MANsL) did not produce higher levels of sLuc than the wild-type plasmid pHPV16ANsL (Figure 5D). In contrast, inactivation of the splicing silencers at SD3632 in tandem with inactivation of splicing silencers at SA5639 as in pM2xmut, resulted in high induction of HPV-16 late gene expression (Figure 5D). The sLuc assay is extremely sensitive, which is why we are able to monitor late gene expression from the HPV-16 genomes. As a comparison, the control plasmid pCMVsluc produced in excess of 1 × 10^6^ sLuc units at 24 h posttransfection (data not shown). These results demonstrated that splicing silencers at both SD3632 and SA5639 inhibited L1 mRNA splicing in the context of the HPV-16 genome and showed that the splicing silencers were active in human primary keratinocytes that were grown in monolayer cultures.

Next we introduced 2-nt substitutions in each AUAGUA motif upstream of SD3632 in pHPV16MANsL, resulting in pME4 (Figure 5C). These substitutions affected the C-terminal sequence of HPV-16 E4, but left E2 intact. The levels of sLuc produced by pME4 were similar to those produced by pM2xmut, demonstrating that 2-nt substitutions in each AUAGUA motif were sufficient to inactivate the splicing silencers at SD3632 (Figure 5D). PCR-analysis of DNA extracted from the human primary keratinocytes transfected with pHPV16ANsL and its derivatives revealed that recombination occurred and episomal HPV-16 DNA formed, as expected (Figure 5E). We concluded that the two AUAGUA motifs at HPV-16 SD3632 were functionally active splicing silencers in human primary keratinocytes grown in monolayer cultures, and that they inhibited HPV-16 late gene expression from the episomal form of the HPV-16 genome.

### Cellular proteins bind directly and specifically to the AUAGUA motifs in the HPV-16 splicing silencer

**Figure 6.**
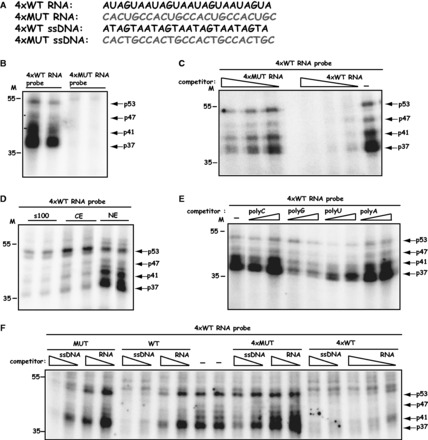
(**A**) Cellular proteins bind directly and specifically to the AUAGUA motifs in the HPV-16 splicing silencer. Sequences of HPV-16 4xWT RNA and 4xMUT RNAs. These RNAs were either used as radiolabeled RNA probes or unlabeled RNA competitors. 4xWT and 4xMUT ssDNAs were used as unlabeled competitors. Mutant RNA or ssDNA sequences are shown in red. (**B**) UV cross-linking of 2-fold serially diluted nuclear extract to *in vitro* synthesized, radiolabeled HPV-16 4xWT or 4xMUT RNA probes. Four bands representing proteins with molecular weights of 37, 41, 47 and 53 kDa were specifically detected with the 4xWT RNA probe. (**C**) UV cross-linking of nuclear extract to *in vitro* synthesized, radiolabeled HPV-16 4xWT RNA probe in the absence or presence of 2-fold serially diluted 4xWT or 4xMUT competitor RNAs. Four bands representing proteins with molecular weights of 37, 41, 47 and 53 kDa were specifically detected with the 4xWT RNA probe. (**D**) UV cross-linking of nuclear extract (NE), cytoplasmic extract (CE) or S100 supernatant obtained after centrifugation of CE for 100 000 *g* (s100) to radiolabeled 4xWT RNA probe. Four bands representing proteins with molecular weights of 37, 41, 47 and 53 kDa were specifically detected with the 4xWT RNA probe. (**E**) UV cross-linking of nuclear extract to *in vitro* synthesized, radiolabeled HPV-16 4xWT RNA probe in the absence or presence of 2-fold serially diluted pC, pG, pU or pA competitor, homoribopolymer RNAs. Four bands representing proteins with molecular weights of 37, 41, 47 and 53 kDa were specifically detected with the 4xWT RNA probe. (**F**) UV cross-linking of nuclear extract to *in vitro* synthesized, radiolabeled HPV-16 4xWT RNA probe in the absence or presence of 2-fold serially diluted, 4xWT or 4xMUT competitor RNAs or ssDNA RNAs. Four bands representing proteins with molecular weights of 37, 41, 47 and 53 kDa were specifically detected with the 4xWT RNA probe.

To investigate whether cellular proteins bind to the AUAGUA motifs, we synthesized radiolabeled RNA consisting of four copies of the wild AUAGUA motifs (4xWT RNA) or four copies of the mutant CACUGC motifs (4xMUT RNA) (Figure 6A). The RNA was subjected to UV cross-linking to nuclear extract from HeLa cells. The results revealed that at least four radiolabeled protein bands were detected after UV cross-linking to 4xWT RNA, but not to 4xMUT RNA (Figure 6B). Duplicate samples are shown, and the proteins were labeled p37, p41, p47 and p53 according to their estimated molecular weights. Cold 4xWT RNA competed efficiently with the radiolabeled probe for all bands, whereas cold 4xMUT RNA competed less well (Figure 6C). The p37, p41 and p47 bands were predominantly nuclear, whereas p53 was found in both nuclear and cytoplasmic fractions (Figure 6D). Competition experiments with cold homoribopolymers revealed that the UV cross-linked proteins had affinity for polyG and polyU in that order, but not for polyC or polyA (Figure 6E). Interestingly, ssDNA oligos of the corresponding 4xWT sequence also competed for the UV cross-linked proteins (Figure 6F) to a similar extant as the 4xWT RNA, whereas equimolar amounts of neither 4xMUT RNA or ssDNA competed efficiently with the radiolabeled probe for the UV cross-linked proteins (Figure 6F). Similarly, the 34-nt wild-type sequence (WT) located upstream of SD3632 competed better than the mutant (MUT) RNA for the cellular proteins binding to the radiolabeled probe (Figure 6F), further supporting the conclusion that cellular proteins interacted with the AUAGUA motifs in a sequence specific fashion, and in a manner that correlated with the splicing inhibitory activity of the various sequences.

### Several members of the hnRNP D family of proteins and hnRNP A2/B1 interact with the HPV-16 splicing silencer

**Figure 7.**
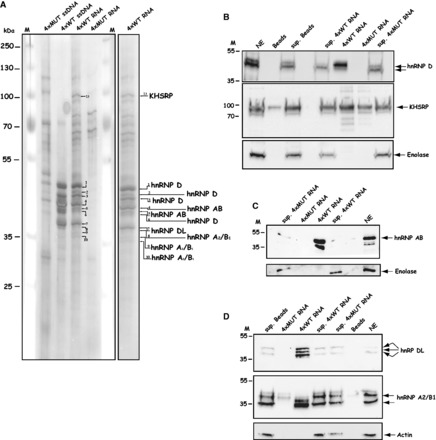
(**A**) Several members of the hnRNP D family of proteins and hnRNP A2/B1 interact with the HPV-16 splicing silencer. RNA-mediated pull down of cellular factors from nuclear extracts using biotinylated 4xMUT or 4xWT ssDNA, or biotinylated 2′-O-Methylated 4xWT or 4xMUT RNA followed by SDS–PAGE and silver staining as described in ‘Materials and Methods’ section. The mass spectrometry result of each excised and eluted band is shown to the right. (**B–D**) Pull down of cellular factors from nuclear extracts using biotinylated 2′-O-methylated 4xWT or 4xMUT RNA followed by western blot analysis with hnRNP D, KHSRP, hnRNP AB, hnRNP DL, hnRNP A2/B1, enolase or actin antibodies. Sup, represents the fraction of unbound proteins located in the supernatant after the pull down; beads, mock pull downs using streptavidine beads in the absence of biotinylated RNA or ssDNA oligonucleotides.

Next we performed RNA protein pull-down experiments from cellular extracts followed by mass spectrometry of individual protein bands detected by the 4xWT RNA and not by the 4xMUT RNA. Multiple silver stained bands were pulled down by the 4xWT RNA, as well as 4xWT ssDNA, but not with the 4xMUT RNA or 4xMUT ssDNA (Figure 7A). The protein bands were identified as hnRNP D, hnRNP DL, hnRNP AB, hnRNP A2/B1 and KHSRP by mass spectrometry. Although hnRNP D, hnRNP DL and hnRNP AB are related and belong to the hnRNP D-family of hnRNP proteins, hnRNP A2/B1 belongs to the hnRNP A-family (48). KHSRP is an unrelated RNA-binding protein. Western blot analysis of proteins pulled down by the 4xWT RNA sequence confirmed that hnRNP D, hnRNP DL, hnRNP AB and hnRNP A2/B1 bound specifically to the 4xWT RNA, whereas KHSRP bound with lower specificity as it was also pulled down by the 4xMUT RNA (Figure 7B–D). KHSRP was therefore not investigated further. Actin or enolase were not pulled down by any of the RNAs and were found unbound in the supernatants from the pull-down experiments (Figure 7B–D). Multiple bands in the silver stained gel represented hnRNP D. Four splice variants of hnRNP D mRNA have been described (Supplementary Figure S4) (49). They either lack exons 2 and 7 or contain one or both (Supplementary Figure S4). Mass spectrometry identified peptides in exon 2, but not in exon 7, suggesting that at least hnRNP D40 was pulled down by the 4xtWT RNA (Supplementary Figure S4), although it does not exclude binding of hnRNP D37 or hnRNP D42. We concluded that the three members of the hnRNP D family of proteins (hnRNP D, hnRNP DL and hnRNP AB) as well as hnRNP A2/B1 interacted specifically with the AUAGUA motif found in the HPV-16 splicing silencer located immediately upstream of late 5′-splice site SD3632. We speculate that one or more of these proteins suppress HPV-16 5′-splice site SD3632.

### Knock-down of the hnRNP D family of proteins or hnRNP A2/B1 induces HPV-16 late gene expression

**Figure 8.**
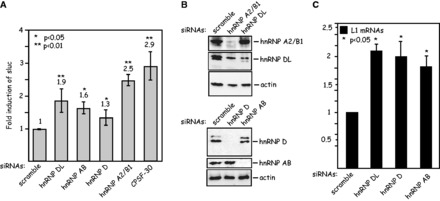
(**A**) Knock-down of the hnRNP D family of proteins or hnRNP A2/B1 induces HPV-16 late gene expression. Fold induction of sLuc activity in the cell culture medium of the C33A2 reporter cell line transfected with the indicated siRNAs. Mean values and standard deviations of six parallel transfections are shown. (**B**) Western blot analysis with antibodies against hnRNP A2/B1, hnRNP DL, hnRNP D, hnRNP AB or actin, on cell extracts from C33A2 cells transfected the indicated siRNAs. (**C**) Real time PCR with HPV-16 L1-specific primers 757s and L1A (for primer location, see Supplementary Figure S5) on cytoplasmic RNA extracted from C33A2 cells transfected with the indicated siRNAs. Statistical *P*-values are indicated.

To investigate whether the identified proteins inhibit HPV-16 late gene expression, we used a previously described reporter assay to measure the effects on HPV-16 late gene expression (36). The cell line C33A2 contains the subgenomic HPV-16 expression plasmid pBELsLuc integrated in the genome (Supplementary Figure S5). The pBELsLuc plasmid encodes HPV-16 early and late genes but expresses only the early mRNAs as HPV-16 late splice sites are suppressed. The HPV-16 L1 gene has been replaced by the poliovirus IRES followed by the sLuc gene and thus functions as a reporter assay for late gene expression as previously described (Supplementary Figure S5) (36). We reasoned that siRNA knock-down of the proteins that bind to the splicing silencer at SD3632 should induce HPV-16 late gene expression if these proteins were suppressing SD3632 in the cervical cancer cells and therefore transfected the C33A2 reporter cell line with siRNAs against all known splice variants of hnRNP D, hnRNP DL, hnRNP AB or hnRNP A2/B1. The results revealed that siRNAs toward these proteins induced HPV-16 late gene expression relative to the control consisting of scrambled siRNA (Figure 8A). The highest induction of sLuciferase was obtained with siRNAs against hnRNP A2/B1, followed by siRNAs against hnRNP DL, hnRNP AB and hnRNP D (Figure 8A). The positive control siRNA against CPSF-30 showed the highest induction of late gene expression as expected, as inhibition of polyadenylation by the HPV E2 protein has been shown previously to induce HPV-16 late gene expression (45). All siRNAs knocked down their corresponding proteins as determined by western blotting, whereas actin levels were unaffected (Figure 8B). We were unable to induce sLuc levels further by mixing siRNAs to various mRNAs owing to toxic effects of the siRNA when higher concentrations were used (data not shown). To provide further support for these results, we also monitored the levels of spliced HPV-16 L1 mRNAs using primers 757s and L1A (se Supplementary Figure S5 for primer locations) following siRNA transfections. As predicted by the sLuc results described earlier in the text, the spliced L1 mRNA levels increased following transfections of the indicator cell line with siRNAs against hnRNP DL, hnRNP AB and hnRNP D compared with cells transfected with scrambled siRNAs (Figure 8C). We concluded that the hnRNP D family of proteins including hnRNP D, hnRNP DL and hnRNP AB, and hnRNP A2/B1 suppress HPV-16 late L1 mRNA splicing in cervical cancer cells.

### Overexpression of hnRNP D/AUF isoforms hnRNP D37 and hnRNP D40 further suppress HPV-16 late gene expression

**Figure 9.**
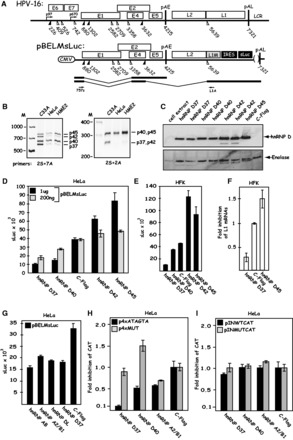
(**A**) Overexpression of hnRNP D/AUF isoforms hnRNP D37 and hnRNP D40 further suppress HPV-16 late gene expression. Schematic representations of the HPV-16 genome and of subgenomic HPV-16 expression plasmid pBELMsLuc. The early E4 mRNA and the late L1 mRNA are shown later in the text. Arrows indicate RT-PCR primers 757s and L1A. (**B**) RT-PCR on cytoplasmic RNA extracted from C33A cells, HeLa cells and HME2 cells (human primary keratinocytes immortalized by HPV-16) using hnRNP D specific primers 2S and 7A or 2S and 2A as indicated. For primer locations on the hnRNP D mRNAs, see Supplementary Figure S4. (**C**) Western blot analysis on cell extracts from HeLa cells transfected with plasmids expressing the various hnRNP D isoforms or the empty vector pC-Flag. The filter was probed with anti-flag antibody. Duplicate transfections are shown. The filter was stripped and probed with antibody against Enolase as a loading control. (**D**) sLuc activity in the cell culture medium of HeLa cells cotransfected with pBELMsLuc and the various hnRNP D expression plasmids and the empty vector pC-Flag. (**E**) sLuc activity in the cell culture medium of primary HFKs cotransfected with pBELMsLuc and the various hnRNP D expression plasmids and the empty vector pC-Flag. (**F**) Fold inhibition of HPV-16 L1 mRNA monitored by qPCR on cytoplasmic RNA extracted from primary HFKs cotransfected with pBELMsLuc and pC-Flag, hnRNP D37 or hnRNP D45. (**G**) sLuc activity in the cell culture medium of HeLa cells cotransfected with pBELMsLuc and the various plasmids expressing hnRNP AB, hnRNP A2/B1, hnRNP DL, hnRNP D37 or empty pC-Flag vector. (**H**) Fold inhibition of of CAT production from p4xATAGTA and p4xMUT cotransfected with pC-Flag (empty vector), hnRNP D37, hnRNP D40 or hnRNP A2/B1 expression plasmid. (**I**) Fold induction of of CAT production from pINWTCAT and pINMUTCAT cotransfected with pC-Flag (empty vector), hnRNP D37, hnRNP D40 or hnRNP A2/B1 expression plasmid.

All members of the hnRNP D family give rise to multiple alternatively spliced mRNAs that produces different forms of each protein (48). hnRNP D, also known as AUF, is the prototype member of the hnRNP D family and alternative splicing generates as many as four different forms of hnRNP D named hnRNP D37, hnRNP D40, hnRNP D42 and hnRNP D45 (50). All four hnRNP D mRNAs are targeted by the siRNA pool used above, and one cannot target single hnRNP D mRNAs specifically owing to the common origin of the mRNAs (Supplementary Figure S4). To investigate the role of the individual hnRNP D proteins in HPV-16 late gene expression, we wished to overexpress each hnRNP D isoform and monitor the effect on HPV-16. In contrast to C33A cells, which express approximately equal amounts of all four hnRNP D isoform mRNAs (Figure 9B), HeLa cells and primary keratinocytes immortalized by HPV-16 (HME2 cells) produced primarily the hnRNP D40 and hnRNP D45 isoforms as a result of retention of exon 2 as determined by RT-PCR (Figure 9B). We therefore performed the overexpression experiments in HeLa cells. Transfected cDNAs encoding flag-tagged hnRNP D proteins hnRNP D37, hnRNP D40, hnRNP D42, hnRNP D45 expressed proteins of the correct sizes, whereas the empty vector pC-Flag did not (Figure 9C). Cotransfection of the hnRNP D isoforms with the subgenomic HPV-16 expression plasmid pBELMsLuc (Figure 9A) revealed that overexpression of hnRNP D37 and hnRNP D40 further suppressed the low levels of HPV-16 late gene expression, whereas hnRNP D42 and hnRNP D45 induced HPV-16 late gene expression in relation to transfection with empty plasmid vector (Figure 9D). Higher levels of transfected hnRNP D37 or hnRNP D40 plasmid displayed a stronger inhibitory effect on HPV-16 late gene expression (compare sLuc produced after transfection with 200 ng or 1 µg of hnRNP D37 or hnRNP D40 plasmid), whereas the opposite was true for hnRNP D42 and hnRNP D45 where higher induction of sLuc was observed with higher levels of transfected plasmid (Figure 9D). The experiment was repeated in primary HFKs with similar results (Figure 9E). A real-time PCR quantitation of the L1 mRNAs in the HFKs transfected with hnRNP D37 and hnRNP D45 confirmed that hnRNP D37 inhibited HPV-16 late gene expression, whereas hnRNP D45 induced HPV-16 late gene expression (Figure 9F). Overexpression of the other proteins binding the silencer (hnRNP AB, hnRNP DL and hnRNP A2/B1) also inhibited late gene expression from pBELMsLuc (Figure 9G). To confirm that the inhibitory effect of hnRNP D37, hnRNP D40 and hnRNP A2/B1 was specific for HPV-16 plasmids containing the wild-type silencer element, plasmids encoding these proteins were cotransfected with p4xATAGTA that contains the wild-type silencer element (see Figure 2) or p4xMUT that contains a mutant silencer element (see Figure 2). hnRNP D37, hnRNP D40 and hnRNP A2/B1 all inhibited expression from the p4xATAGTA plasmid (Figure 9H). hnRNP D37 and hnRNP D40 did not inhibit expression from the mutant plasmid p4xMUT, demonstrating a specific inhibition of the wild-type silencer, whereas hnRNP A2/B1 also showed inhibition of the mutant plasmid p4xMUT, suggesting that hnRNP A2/B1 also affected other HPV-16 sequences on the reporter plasmids. In stark contrast, none of the proteins inhibited expression from plasmids expressing ‘pre-spliced’ HPV-16 mRNAs, irrespectively of the presence of wild-type or mutant silencer elements (Figure 9I). For structures of plasmids pINWTCAT and pINMUTCAT, see Supplementary Figure S3. These results supported the idea that the hnRNP D proteins D37 and D40 and hnRNP A2/B1 inhibit splicing at HPV-16 late splice site SD3632.

## DISCUSSION

Two HPV-16 splice sites are used exclusively to produce spliced L1 mRNAs: SD3632 and SA5639 (17,19). Here we show that hnRNP D proteins and hnRNP A2/B1 inhibit SD3632. We have previously shown that the late 3′-splice site SA5639 is controlled by splicing silencers located in the L1 coding region downstream of SA5639, and that these silencers bind hnRNP A1 (19,29,30). In contrast, both hnRNP A1 and hnRNP A2/B1 stimulate splicing of early HPV-16 mRNAs thereby enhancing production of E7 mRNAs (51). High levels of hnRNP A1 and hnRNP A2/B1 therefore inhibit HPV-16 late gene expression and enhance HPV-16 early mRNA expression. HPV-16 L1 production shows a typical differentiation dependent expression pattern in the squamous epithelium and is detected exclusively in cells located at the very top of the epithelium (9–11). In contrast, hnRNP A1, hnRNP A2/B1 and the hnRNP D proteins (those for which the expression profile in the cervical epithelium is known) are highly expressed in the low-to-mid layers of the epithelium, with little or no expression in the terminally differentiated cells at the top of the epithelium (www.proteinatlas.org). The hnRNP A1, hnRNP A2/B1 and the hnRNP D proteins are also highly expressed in cervical cancer cells (www.proteinatlas.org). Therefore, there is an inverse correlation between hnRNP A1, hnRNP A2/B1 and hnRNP D protein production in relation to the HPV-16 L1 protein production, which supports an inhibitory role of the proteins in HPV-16 L1 expression.

The hnRNP D gene expresses four alternatively spliced mRNAs (49). Although hnRNP D37 lacks exons 2 and 7, hnRNP D40 contains only exon 2, hnRNP D42 only exon 7 and hnRNP D45 contains both. Previously published work has shown that they have distinct properties, e.g. some isoforms stabilized labile cellular mRNAs, whereas others did not (52) and hnRNP D42 and hnRNP D45 could activate transcription while hnRNP D37 and hnRNP D40 could not (53). We show that knock-down of all hnRNP D proteins in C33A cells induced HPV-16 late gene expression. Overexpression of hnRNP D37 and hnRNP D40 further suppressed HPV-16 late gene expression, whereas hnRNP D42 and hnRNP D45 activated late gene expression. These hnRNP D isoform-specific effects suggest that increasing hnRNP D37 and D40 levels further enhances suppression of HPV-16 late gene expression, and that hnRNP D42 and 45 interfere with suppression, perhaps by interfering with the function of endogenous hnRNP D proteins. The hnRNP D proteins can be posttranslationally modified by methylation in the C-terminus (54) and by phosphorylation (55) and ubquitination (56) in an isoform-specific manner. The presence or absence of exons 2 and 7 and/or the posttranslational modifications of the various hnRNP D isoforms may affect their ability to form heterodimers and to bind RNA or may alter their subcellular localization or ability to interact with other cellular factors (57,58). The isoform-specific effect of hnRNP D on HPV-16 late gene expression adds to the complexity of HPV-16 gene regulation. However, this is not without precedent as overexpression of the hnRNP D37 and D40 isoforms suppressed production of the HIV-1 gag capsid protein, whereas overexpression of hnRNP D42 and D45 increased it (59). Similarly, we found that hnRNP D37 and D40 isoforms suppressed production of the HPV-16 L1 mRNA, whereas overexpression of hnRNP D42 and D45 caused an induction of the same mRNA. It has been shown previously that the isoforms are differently expressed in some tissues (49). It will be interesting to investigate whether these levels change in response to epithelial cell differentiation or progression to malignancy through premalignant cervical lesions. Interestingly, a cell line generated by immortalization of primary human keratinocytes by HPV-16 produced primarily hnRNP D40 and hnRNP D45 mRNAs, as did the HPV-18 positive HeLa cells. In contrast, HPV-negative cervical cancer cell line C33A expressed relatively similar amounts of all four alternatively spliced hnRNP D mRNAs. Therefore, HPV infection may alter hnRNP D mRNA splicing.

The hnRNP D proteins act primarily by enhancing decay of labile mRNAs that encode AU-rich RNA elements in their 3′-UTR (58). hnRNP D binds other RNA and ssDNA sequences specifically, such as the pre-mRNA 3′ splice site sequence r(UUAG/G) and the human telomeric DNA sequence d(TTAGGG)n (60) and has affinity for polyG, or to AUAGUA and ATAGTA as described herein. However, hnRNP D may also stabilize mRNAs (61) and play a role in mRNA translation (62) transcription (53) and splicing (60). Our results suggest that hnRNP D proteins inhibit the major late 5′-splice site of HPV-16. Similarly to hnRNP A1 (63), which cooperatively binds to RNA to inhibit splice sites, it has been shown that hnRNP D can bind RNA in a cooperative manner (57). One may speculate that cooperative binding of some of the hnRNP D isoforms to the splicing silencer of HPV-16 SD3632 causes inhibition of this splice site. Less is known about hnRNP DL (64), also known as hnRPD-like protein and JKT41-binding protein. Similarly to hnRNP D, it has several isoforms and has affinity for polyG. It binds to AU-rich RNA instability elements in TNF-alpha and COX-2 mRNAs (64) and may regulate translation of human nuclear factor kappa B-repressing factor (65). Our results suggest that hnRNP DL may also be involved in regulation of splicing. hnRNP AB on the other hand has been found in prespliceosomal complexes supporting the idea of a role in splicing regulation as suggested by our results (66). hnRNP A2/B1 binds to RNA trafficking elements (67) and to recently identified RNA stability elements (68) and may therefore contribute to many steps in cellular RNA processing pathways. However, the hnRNP A2/B1 protein is best known as a splicing factor that binds to splicing silencers and inhibits splicing to both 5′- and 3′-splice sites (69-71), similarly to its effect on HPV-16 late 5′-splice SD3632 proposed here.

Despite the fact that hnRNP D suppresses expression of many cell cycle regulatory factors by promoting degradation of their mRNAs (57), overexpression of hnRNP D has also been shown to prevent cellular senescence (53) and to lead to tumorigenesis (72). Expression of hnRNP A2/B1 protein is deregulated and overexpressed in many different cancer forms and has been shown to drive tumorigenesis (73) and control invasive cell migration (74) and epithelial-mesenchymal transition (75). This is interesting as high levels of hnRNP A2/B1 enhances splicing of the HPV-16 early pre-mRNAs to generate E7 mRNAs and inhibits HPV-16 late mRNAs splicing, thereby creating the HPV-16 gene expression profile seen in HPV-16 high grade lesions or cancers in which the HPV-16 genome has not integrated into the host cell chromosomes. In contrast, downregulation of hnRNP A1, hnRNP A2/B1 and the hnRNP D proteins may be a prerequisite for entry into the late stage of the HPV-16 infection during a productive infection.

Genital HPV infections are common in the human population, and in terms of transmission, HPVs are successful viruses. A subset of the genital HPV types is termed high-risk HPV types as a result of its association with cancer, primarily cervical cancer in women. HPV-16 sticks out as the most commonly found HPV type in the human population as well as in cervical cancer, probably due to enhanced capability to establish persistence in addition to its well-defined role in cell transformation (1,6). From the results presented in this manuscript, it appears that sequences upstream of the unique late 5′-splice site derived from different HPV types differ in their ability to inhibit splicing. Of all HPVs tested here, one from each genus that contains at least one HPV, all HPV types inhibited L1 mRNA splicing less well than HPV-16. Although it may be argued that the various HPV sequences were all tested on HPV-16 SD3632, the difference in ability of the various HPV types to inhibit HPV late mRNA splicing is great. The identified splicing inhibitory motif AUAGUA is repeated twice in the HPV-16 splicing silencer sequence. However, it is not conserved in the other HPV types. HPV-18 contains two, and HPV-6 one related motif, but they do not appear to inhibit splicing efficiently. Therefore, the presence of AUAGUA splicing silencer sequence is unique to HPV-16. One may speculate that efficient inhibition of late gene expression correlates with ability to avoid detection by the immune system and establish viral persistence.

The functional analysis of the splicing silencer elements at HPV-16 SD3632 and SA5639 in the context of the full-length HPV-16 genome, as well as in primary human keratinocytes, established that these RNA elements are important in the HPV-16 gene expression program. The mutations introduced in pME4 affected the E4 amino acid sequence, but not E2 (Figures 4 and 5C). Four amino acids at the end of E4 were altered, and three amino acids were added to the E4 protein as the E4 stop codon was affected by the mutations (Figure 4). These mutations in E4 could potentially affect E4 protein function, and thereby indirectly late gene expression. However, previous results with E4 knock-out mutants in genomic clones of HPV-16 (76) or HPV-31 (77) genomes revealed a phenotype at later stages in the viral life cycle, in response to differentiation. We have used a short term, transient transfection assay with proliferating keratinocytes, in the absence of cell differentiation. In addition, the E4 mutants described previously, displayed a reduction in late gene expression (76,77), whereas our mutants showed enhanced late gene expression (Figure 5D). We do not find it likely that the phenotype of the splicing silencer mutants described here is a result of the altered E4 protein sequence. It would therefore be of great interest to study the effects of these mutations on the HPV-16 gene expression profile in a differentiating environment.

## SUPPLEMENTARY DATA

Supplementary Data are available at NAR Online.

## FUNDING

Funding for open access charge: Swedish Research Council-Medicine [2012-1933]; Swedish Cancer Society [CAN 2012/542].

*Conflict of interest statement*. None declared.

## Supplementary Material

Supplementary Data
