# Supplementary material for: Suppression of HPV-16 late L1 5′-splice site SD3632 by binding of hnRNP D proteins and hnRNP A2/B1 to upstream AUAGUA RNA motifs
Source: Nucleic Acids Res. 2013 Sep 5;41(22):10488–508. doi: 10.1093/nar/gkt803 (PMC3905901; doi:10.1093/nar/gkt803)
Supplement: Supplementary Data [file supp_41_22_10488__index.html]

Suppression of HPV-16 late L1 5′-splice site SD3632 by binding of hnRNP D proteins and hnRNP A2/B1 to upstream AUAGUA RNA motifs — Suppression of HPV-16 late L1 5′-splice site SD3632 by binding of hnRNP D proteins and hnRNP A2/B1 to upstream AUAGUA RNA motifs — Supplementary Data 

# Suppression of HPV-16 late L1 5′-splice site SD3632 by binding of hnRNP D proteins and hnRNP A2/B1 to upstream AUAGUA RNA motifs

## Supplementary Data

files

**Files in this Data Supplement:**

- Supplementary Data - pdf file
