# Supplementary material for: Suppression of HPV-16 late L1 5′-splice site SD3632 by binding of hnRNP D proteins and hnRNP A2/B1 to upstream AUAGUA RNA motifs
Source: Nucleic Acids Res. 2013 Sep 5;41(22):10488–508. doi: 10.1093/nar/gkt803 (PMC3905901; doi:10.1093/nar/gkt803)
Supplement: Supplementary Data [file supp_gkt803_nar-01110-a-2013-File011.pdf]

Figure S1.

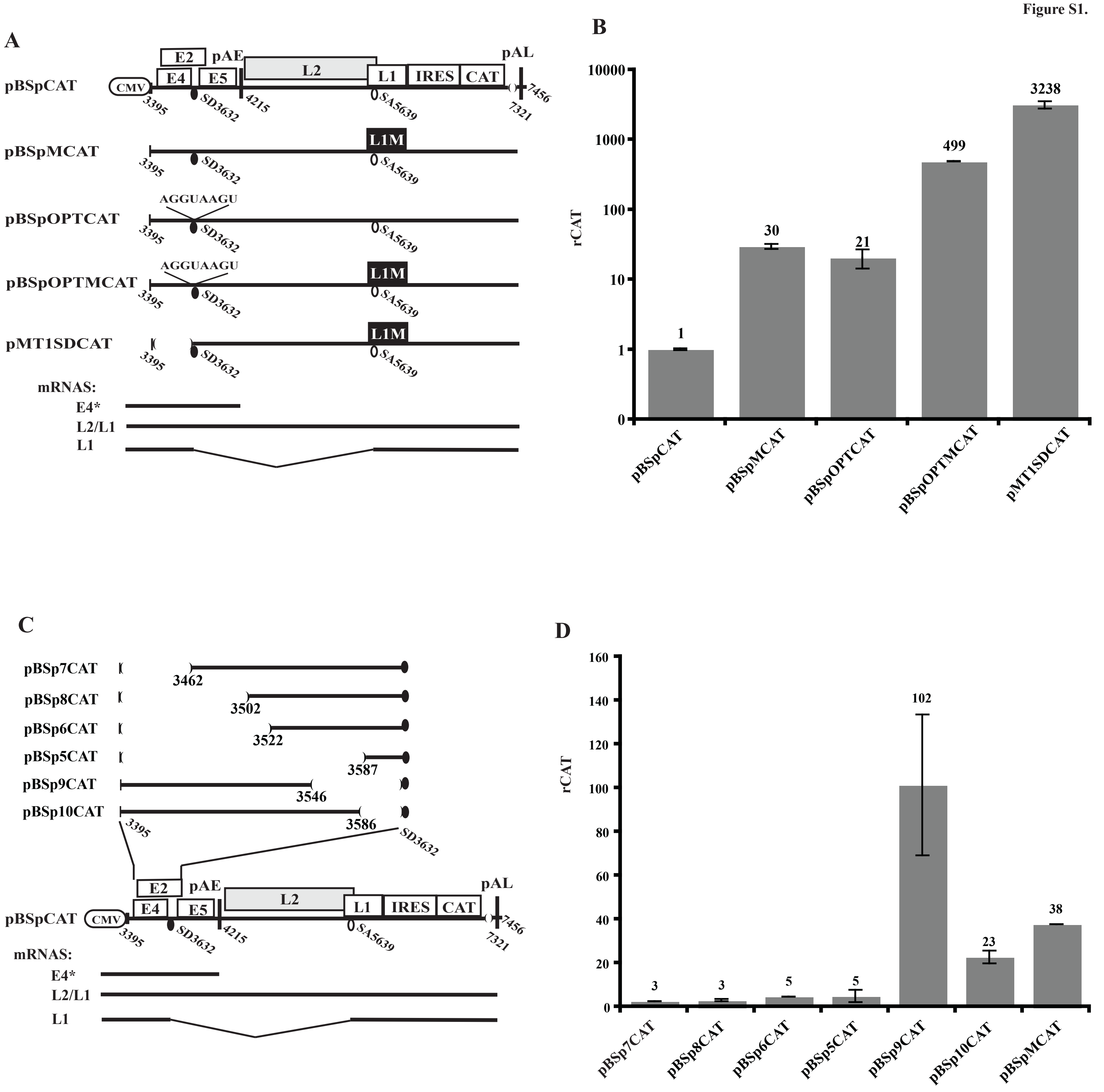
A
pBSpCAT
CMV
E2
E4
E5
pAE
L2
L1
IRES
CAT
pAL
3395
SD3632
4215
SA5639
7321
7456
pBSpMCAT
L1M
pBSpOPTCAT
AGGUAAGU
pBSpOPTMCAT
pMT1SDCAT
mRNAS:
E4*
L2/L1
L1
B
rCAT
10000
1000
100
10
1
0
1
30
21
499
3238
pBSpCAT
pBSpMCAT
pBSpOPTCAT
pBSpOPTMCAT
pMT1SDCAT
C
pBSp7CAT
3462
pBSp8CAT
3502
pBSp6CAT
3522
pBSp5CAT
3587
pBSp9CAT
3546
pBSp10CAT
3586
D
rCAT
160
140
120
100
80
60
40
20
0
3
3
5
5
102
23
38
pBSp7CAT
pBSp8CAT
pBSp6CAT
pBSp5CAT
pBSp9CAT
pBSp10CAT
pBSpMCAT

Figure S2.

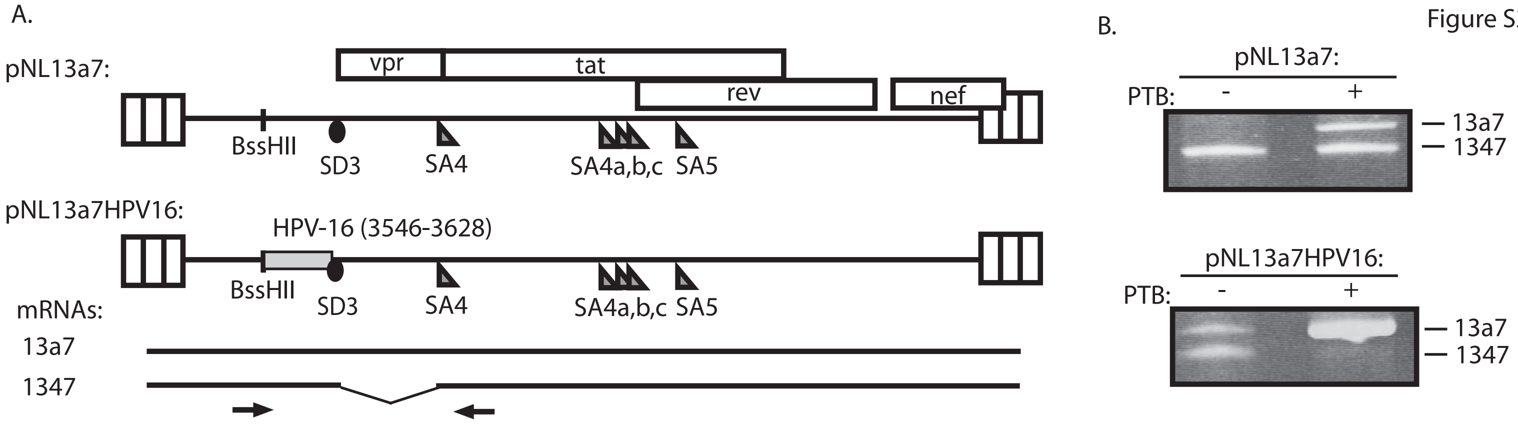
A.
pNL13a7:
vpr
tat
rev
nef
BssHII
SD3
SA4
SA4a,b,c
SA5
pNL13a7HPV16:
HPV-16 (3546-3628)
BssHII
SD3
SA4
SA4a,b,c
SA5
mRNAs:
13a7
1347
B.
pNL13a7:
PTB:
-
+
13a7
1347
pNL13a7HPV16:
PTB:
-
+
13a7
1347

Figure S3.

| | 3599 … SD3632 | type | species | genus | rCAT |
|---|---|---|---|---|---|
| pBSpD1MCAT | AATAGTAACACTACACCCATAGTACATTTAAAAGGT | 16 | 9 | Alpha | 50 ± 1.9 |
| pHPV6MCAT | AACAGTTCAGCTACGCCTATAGTGCAATTTCAAGGT | 6 | 10 | Alpha | 1019 ± 82 |
| pHPV18MCAT | AGTGGTAACACTACGCCTATAATACATTTAAAAGGT | 18 | 7 | Alpha | 1018 ± 157 |
| pHPVMCAT | TGTTGTAAAACTACGCCTATAGTACATTTAAAAGGT | Cons. | (7,9,10) | Alpha | 114 ± 26 |
| pHPV41MCAT | CCAAAGGTCCAGTGAATAGCCTGCGGTGCTTAAGGT | 41 | 1 | Nu | 900 ± 114 |
| pHPV1MCAT | TAAAAGGGGGTGCCAATCAGCTTAAGTGTCTCAGGT | 1 | 1 | Mu | 174 ± 11 |
| pHPV4MCAT | GGAGCAGACATCGACAAGTTGAAAGACAAGGTCTGT | 4 | 1 | Gamma | 1491 ± 201 |
| pHPV5SMCAT | AACAGCCACAACAAACCGAAACCAGAGGAAGAAGGT | 5 | 1 | Beta | 999 ± 178 |
| pHPV5LMCAT | ACACCACTCGGTCCACCACCAGGTCCCGGTCCACGT | 5 | 1 | Beta | 104 ± 20 |

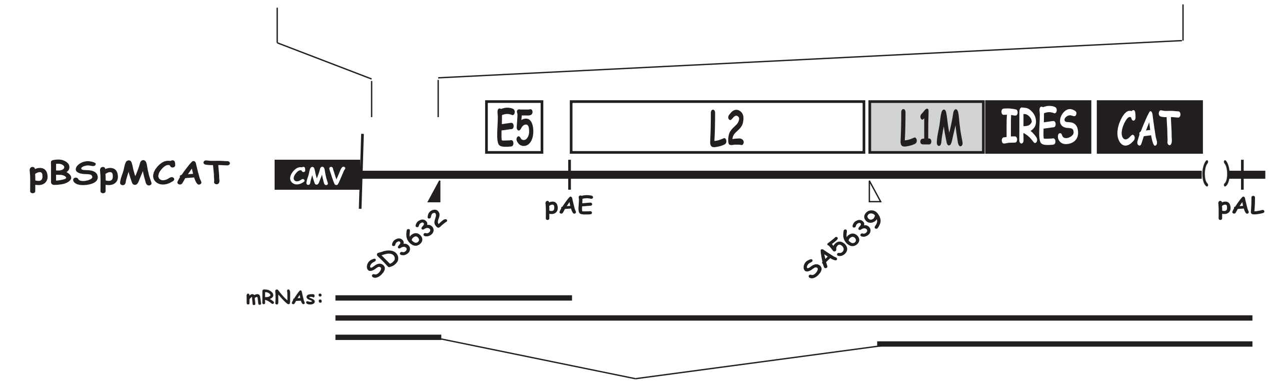

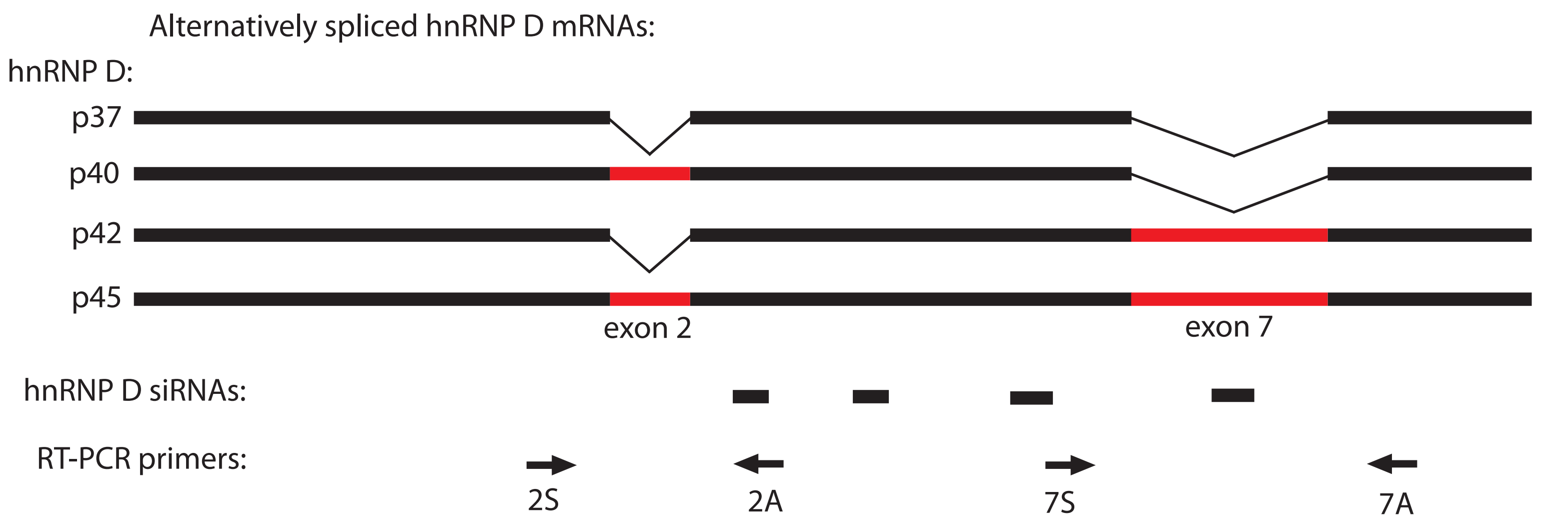
Alternatively spliced hnRNP D mRNAs:
hnRNP D:
p37
p40
p42
p45
exon 2
exon 7
hnRNP D siRNAs:
RT-PCR primers:
2S
2A
7S
7A

# C33A2 reporter cell line for HPV-16 late gene expression:

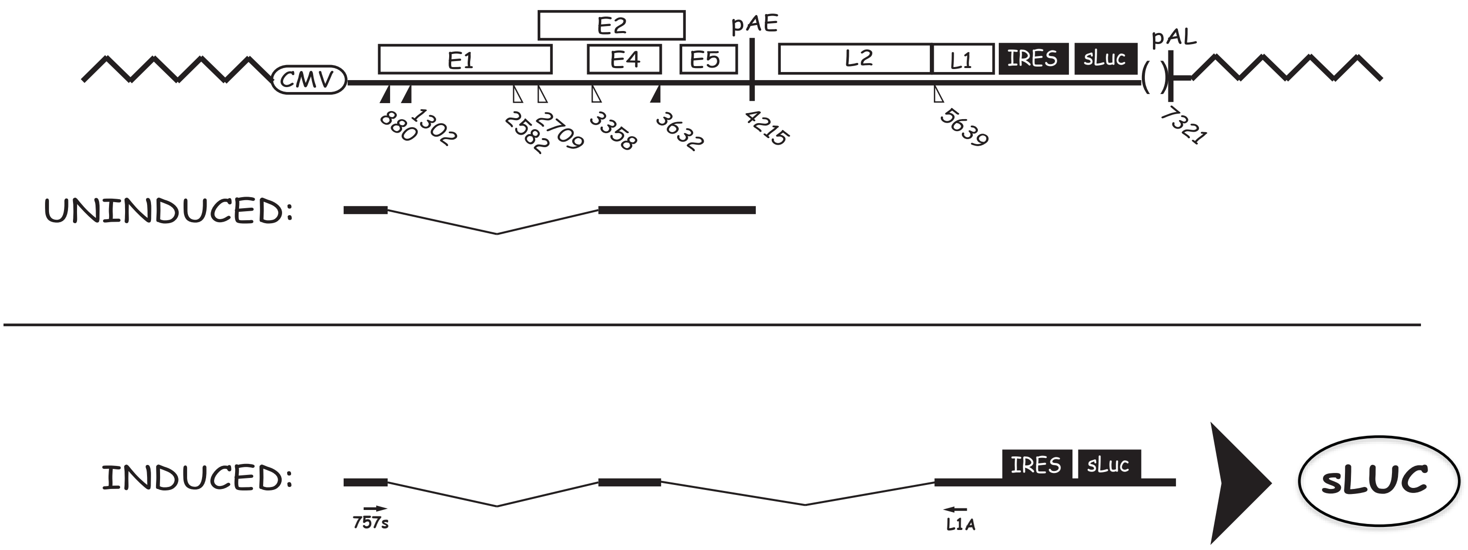

**Supplemental figure legends.**

**Fig. S1 (A). HPV-16 late splice sites SD3632 and SA5639 are suboptimal.** Schematic representation of subgenomic HPV-16 expression plasmids. Numbers indicate nucleotide positions of 5'- (filled circles) and 3'-splice sites (open circles) or the early and late poly (A) signals pAE and pAL, respectively, and the border the HPV sequences. L1M, a mutant L1 sequence in which splicing silencers downstream of SA5639 had been inactivated; IRES, the poliovirus internal ribosome entry site sequence; CAT, CAT reporter gene; CMV, human cytomegalovirus immediate early promoter. mRNAs produced by the HPV-16 plasmids are indicated. The sequence of the optimized 5'-splice site SD3632 is indicated. **(B)** CAT protein levels produced in transfected cells. rCAT was calculated as described in Materials and Methods. Mean values and standard deviations are shown. Note the logarithmic scale. **(C)** Schematic representation of subgenomic HPV-16 expression plasmid pBSpCAT with various deletions. Numbers indicate nucleotide positions of 5'- (filled circles) and 3'-splice sites (open circles) or the early and late poly (A) signals pAE and pAL, respectively, and the borders of deletions. **(D)** CAT protein levels produced in transfected cells. rCAT was calculated as described in Materials and Methods. Mean values and standard deviations are shown.

**Fig. S2. The splicing silencer of HPV-16 SD3632 functions in a heterologous context. (A)** The sequence immediately upstream of HPV-16 SD3632 (nt 3546-3628) was inserted immediately upstream of HIV-1 5'splice site SD3 in the HIV-1 vpr cDNA expression plasmid pNL13a7. **(B)** The effect of the HPV-16 splicing silencer on the splicing from HIV-1 SD3 to SA4 was monitored by RT-PCR. Overexpression of the splicing inhibitory factor PTB was performed to show production of the unspliced 13a7 vpr mRNA from pNL13a7.

**Fig. S3. The presence of a splicing silencer immediately upstream of the unique HPV late 5'-splice site is not conserved among HPVs.** Schematic representations of subgenomic HPV expression plasmids. Plasmid names are shown to the left. HPV sequences present upstream of HPV-16 SD3632 in each plasmid are shown. The ATAGTA and ACAC motifs are underlined. The type, species and genus of each human papillomavirus sequence inserted in the subgenomic HPV-16 CAT plasmid are listed to the right (45). Cons. represents a consensus sequence of all known HPV species 7, 9 and 10 of the alphavirus genus. Filled and empty triangles represent HPV-16 late 5'-splice site SD3632 and late 3'-splice site SA5639. Early and late poly (A) signals pAE and pAL are indicated. L1M, a mutant L1 sequence in which splicing silencers downstream of SA5639 had been inactivated (29,32); CMV, human cytomegalovirus immediate early promoter; IRES, the poliovirus internal ribosome entry site sequence; CAT, CAT reporter gene. mRNAs produced by the HPV plasmids are indicated. CAT protein levels produced by each plasmid in the transfected cells are shown to the right. rCAT was calculated as described in Materials and Methods. Mean values and standard deviations are shown.

**Fig. S4. All four alternatively spliced hnRNP D mRNAs are targeted by siRNAs.** Schematic representation of the alternatively spliced hnRNP D mRNAs. Black bars represent constitutively spliced exons while red bars represent the alternatively spliced exons 2 and 7. Short black bars represent the siRNAs present in the siRNA pool directed towards the hnRNP D mRNAs. Arrows indicated the PCR primers used for RT-PCR amplification of hnRNP D cDNAs.

**Fig. S5. Reporter cell line C33A2 contains a subgenomic HPV-16 expression plasmid from which induction of late gene expression can be monitored as an increase in extracellular sLuc activity.** Schematic representation of the subgenomic HPV-16 expression plasmid pBELsLuc stably integrated into the genome of the C33A2 reporter cell line. Numbers indicate nucleotide positions of 5'- (filled triangles) and 3'-splice sites (open triangles) or the early and late poly (A) signals pAE and pAL, respectively. IRES, the poliovirus internal ribosome entry site sequence; sLuc, secreted luciferase reporter gene; CMV, human cytomegalovirus immediate early promoter. mRNAs produced by HPV-16 plasmids are indicated. The uninduced reporter cell line produces primarily E4 mRNA due to suppression of late splice sites SD3632 and SA5639 as well as efficient polyadenylation at pAE. Activation of late mRNA splice sites SD3632 or SA5639 activates production of the spliced late L1 mRNA than produces secreted Luciferase. Spliced L1 mRNAs can also be monitored by RT-PCR using the indicated primers 757s and L1A.
